# Elucidation of miRNAs-Mediated Responses to Low Nitrogen Stress by Deep Sequencing of Two Soybean Genotypes

**DOI:** 10.1371/journal.pone.0067423

**Published:** 2013-07-08

**Authors:** Yejian Wang, Chanjuan Zhang, Qinnan Hao, Aihua Sha, Rong Zhou, Xinan Zhou, Longping Yuan

**Affiliations:** 1 Long Ping Branch, Graduate School of Central South University, Changsha, China; 2 Key Laboratory of Oil Crop Biology of the Ministry of Agriculture, Oil Crops Research Institute of the Chinese Academy of Agricultural Sciences, Wuhan, China; 3 National Hybrid Rice R&D Center, Changsha, China; 4 Graduate School of Chinese Academy of Agricultural Sciences, Beijing, China; 5 Institute of Crops Research, Hunan Academy of Agricultural Sciences, Changsha, China; University Paris South, France

## Abstract

Nitrogen (N) is a major limiting factor in crop production, and plant adaptive responses to low N are involved in many post-transcriptional regulation. Recent studies indicate that miRNAs play important roles in adaptive responses. However, miRNAs in soybean adaptive responses to N limitation have been not reported. We constructed sixteen libraries to identify low N-responsive miRNAs on a genome-wide scale using samples from 2 different genotypes (low N sensitive and low N tolerant) subjected to various periods of low nitrogen stress. Using high-throughput sequencing technology (Illumina-Solexa), we identified 362 known miRNAs variants belonging to 158 families and 90 new miRNAs belonging to 55 families. Among these known miRNAs variants, almost 50% were not different from annotated miRNAs in miRBase. Analyses of their expression patterns showed 150 known miRNAs variants as well as 2 novel miRNAs with differential expressions. These differentially expressed miRNAs between the two soybean genotypes were compared and classified into three groups based on their expression patterns. Predicted targets of these miRNAs were involved in various metabolic and regulatory pathways such as protein degradation, carbohydrate metabolism, hormone signaling pathway, and cellular transport. These findings suggest that miRNAs play important roles in soybean response to low N and contribute to the understanding of the genetic basis of differences in adaptive responses to N limitation between the two soybean genotypes. Our study provides basis for expounding the complex gene regulatory network of these miRNAs.

## Introduction

Nitrogen (N) is an essential macronutrient of plants and its availability markedly affects crop growth and development [1]. The production of high-yielding crops always demands application of substantial N fertilizer [2]. However, due to incomplete capture and poor conversion of N fertilizer, 50–70% of N fertilizer is lost, which results in serious environmental pollution [3].Thus, lowering fertilizer input and improving N use efficiency of crops is urgent for improvement of current agricultural practice [4]. Studying the biological basis of the response of crops to low N is an essential step towards improving their N use efficiency. Several studies have been undertaken to decipher the response of plants to low N, and these studies elucidate the physiological and biochemical changes that are specifically involved in the response. These include the reduction of growth and photosynthesis, remobilization of N from old mature organs to actively growing ones, and accumulation of abundant anthocyanins [5]–[11]. Furthermore, the expression of many plant genes, such as those involved in N absorption and assimilation, carbon metabolism, photosynthesis anthocyanin synthesis, and protein degradation were found to be regulated by N limitation [12]. These studies had provided valuable insights into the plants response to N limitation; however, the mechanisms of responses are far from being completely understood. Recently, some miRNAs have been associated with nutrients limitation in plant, which further facilitate in understanding the adaptability of plants to N limitation [13]–[17].

miRNAs, as a class of non-coding small RNAs (20∼24 nt), are widespread in both plants and animals [18]–[20]. They are derived from primary transcripts that are capable of forming characteristic stem-loop structures and regulate plant gene expression by direct cleavage of their target transcripts or translational repression [18]. The biological functions of miRNAs were believed to mainly involve the regulation of plant growth and development [21]–[25]. However, these small RNAs have emerged as important participants in the plant’s adaptive responses to diverse environmental stresses [13]–[17], [26], [27], for example, miR395, miR398, and miR399 respond to sulfur (S), copper (Cu), and Pi deficiency, respectively. Interestingly, miRNAs were also found to be involved in the plant’s response to N availability. For example, miR167 was found to mediate a pericycle specific response to N [28]. Under N limitation, several pri-miR169 species as well as pri-miR398a decreased in Arabidopsis seedlings, whereas several pri-miR156 species and pri-miR447c were found to be induced [29]. Nine miRNA families (miR164, miR169, miR172, miR397, miR398, miR399, miR408, miR528, and miR827) and nine miRNA families (miR160, miR167, miR168, miR169, miR319, miR395, miR399, miR408, and miR528) were identified to respond to low N in maize shoots and roots respectively [30]. These miRNAs displayed different expression patterns in response to low N in different crops [28]–[30].

miRNAs were initially identified through direct cloning and computational analysis [31]–[34], and most of them are of high abundance and highly conserved [35]. The advent of high-throughput sequencing technology has provided opportunity for the large-scale identification of low abundance miRNAs, thus rapidly increasing the total number of identified plant miRNAs [36]–[37]. Moreover, due to its reproducibility and quantitative feature, high-throughput sequencing can also be used to study the differential expression of miRNAs [38]. Up to now, the technology has been used to identify miRNAs in a large variety of plants such as Arabidopsis, rice, poplar, wheat and tomato [38], [39]–[41].

Soybean (*Glycine max (L.) Merrill*), the major legume crop worldwide, is an important protein source and economic crop. Although soybean can acquire N via its N-fixing symbiosis with rhizobacteria, exogenous N fertilizer is still applied to meet the demand of soybean growth, especially in high-yield production [42], so improving the N use efficiency of soybean is very important. Many miRNAs have been identified in soybean by both computational analysis and high-throughput sequencing [43]–[48]. However, none of these miRNAs were found to be associated with low N stress.

To identify low N-responsive miRNAs in soybean on a genome-wide scale, we constructed 16 small RNAs libraries using samples from 2 different genotypes (low N sensitive and low N tolerant) subjected to various periods of low nitrogen stress. Through high-throughput sequencing and analysis, a total of 362 known miRNAs variants of 158 families and 90 new miRNAs of 55 families were obtained. Moreover, we also found that some soybean miRNAs showed differential expression patterns in response to low N stress. Some potential targets of these miRNAs were predicted to be involved in different biological functions. To the best of our knowledge, this is the first report of systematic investigation of low N -regulated miRNAs and their targets in soybean.

## Materials and Methods

### Plant Materials, Stress Treatments and Sampling

Two soybean cultivar, No.116 (low-N-tolerant soybean variety) and No.84-70 (low-N-sensitive soybean variety) were selected for this study [49]. Seeds of the two varieties were germinated and grown hydroponically with half-strength modified Hoagland solution (7.5 mM/L N) in the greenhouse as previously described [49], The nutrient solution was replaced with fresh solution every 2 days. After they had grown for 10 days until the first trifoliate leaves fully developed, seedlings were transferred to 1/10 N concentrations (0.75 mM/L N) half-strength Hoagland solution for different term (short-term: 0.5 h, 2 h, 6 h,12 h and long-term: 3 d, 6 d, 9 d, 12 d) low N stress treatment. Ca and K were compensated with CaCl_2_ and K_2_SO_4_ respectively at equivalent concentration in low-N Hoagland solution. Control treatment seedlings were maintained in normal N level half-strength Hoagland solution. Each treatment was replicated for thrice. Roots and shoots of total fifteen seedlings with different time point treats were sampled separately, immediately frozen in liquid nitrogen frozen in liquid N and stored at −80°C for RNA extraction. Control treatment samples were collected at the same time.

### Small RNA Library Construction and Sequencing

Small RNA isolation and library construction were carried out as described by Hafner et al [50].Total RNAs were extracted separately from above samples [2 genotypes (No.116, low N-tolerant variety; No. 84-70, low N-sensitive variety) × 2 treats methods (low N-stress and control) × 2 tissues (roots and shoots) × 8 time points [short-term (0.5 h, 2 h, 6 h,12 h) and long-term (3 d, 6 d, 9 d, 12 d)] using Trizol kit (Invitrogen, USA). The quality and integrity of total RNA was analyzed using Agilent 2100. Equal amounts (5 µg) of total RNA from short-term (0.5 h, 2 h, 6 h, 12 h) were pooled as short-term library, and equal amounts (5 µg) of total RNA from long-term (3 d, 6 d, 9 d, 12 d) were pooled as long-term library. Thus, sixteen small RNA libraries were constructed: 116RS, (116-root short-term treatment); 116RL, (116-root long-term treatment); 84RS, (84-70-root short-term treatment); 84RL, (84-70-root long-term treatment); 116SS, (116-shoot short-term treatment); 116SL, (116-shoot long-term treatment); 84SS, (84-70-shoot short-term treatment); 84SL, (84-70-shoot long-term treatment); 116RSC, (116-root short-term control); 116RLC, (116-root long-term control); 84RSC, (84-70-root short-term control); 84RLC, (84-70-root long-term control);116SSC, (116-shoot short-term control); 116SLC, (116-shoot long-term control); 84SSC, (84-70-shoot short-term control); 84SLC, (84-70-shoot long-term control). The Solexa/Illumina sequencing was performed at Beijing Genomics Institute (BGI, Shenzhen, China). Briefly, total RNAs were separated on 15% denaturing PAGE for 10–30 nt small RNAs selection and then ligated with Solexa 5′ and 3′ adapters sequentially. After ligation and purification, adapter-ligated small RNAs were reverse transcribed and 15-cycles pre-amplified, and PCR products were sequenced using Illumina HiSeq 2000.

### Analysis of Sequencing Data

The raw data from Solexa sequencing were preprocessed to remove contaminant reads and clip adapter sequences, and the adapter-trimmed reads longer than 18 nt were used for further analysis as clean reads. The identical clean reads were grouped as unique sequences with associated counts of the individual reads, and each unique sequence was then mapped to the soybean genome *(* http://www.phytozome.net/search.php?show=text&method=Org_Gmax *)* using SOAP v1.11 and no mismatches were allowed [51]. The unique RNA sequences that perfectly matched soybean genome were retained for subsequent analysis.

To identify known miRNAs, those matched-genome unique RNA sequences were aligned with soybean stem-loop miRNA precursors from miRBase 19 *(* http://www.mirbase.org *)*. Generally, in Solexa sequencing, many variants can also map on miRNA precursors besides annotated miRNA sequences, and the reads of variants are less abundant than those of annotated miRNA sequences. However, some research found that variants are more abundant in some cases, indicating that they could be utilized to refine the annotated miRNA sequences in the miRBase [52]–[54]. In our study, owing to the construction of 16 small RNA libraries, variants could be compared among libraries. if a variant was more abundant than the annotated miRNA sequences in most libraries, the variant could be convincingly used to substitute for the annotated miRNA sequences. Thus, the most abundant variants in each library were determined from the comparative analysis.

For novel miRNA predictions, these unique RNA sequences matched to known miRNAs precursors, rRNA etc deposited at GenBank *(* http://www.ncbi.nih.gov/GenBank/ *)* and Rfam *(* http://rfam.sanger.ac.uk/ *)* databases and genomic exon sequences in the sense strand were removed. 100 nt upstream and 100 nt downstream genome sequences flanking the remaining sequences were extracted to predict secondary structures using RNAfold [55], the resulting potential loci with good hairpin-like structures were then analyzed to predict novel miRNAs by Mireap (http://sourceforge.net/projects/mireap/). Parameters were set based on authentic criteria for annotation of plant miRNAs [56].

Solexa data have been deposited into the NCBI database with accession number SRP021551.

### Identifying miRNAs Responsive to low N Stress

In order to identify low N stress responsive miRNAs, the differentially expressed miRNAs(Known miRNAs and new miRNAs)between low-N stress library and corresponding control treatment library need to be investigated. The frequency of miRNA read counts was first normalized as transcripts per million (TPM), then the normalized expression levels of miRNA between the low-N stress and corresponding control samples was carried out to calculate fold change based on the following formula: Fold-change = log_2_ (stress/control).If the normalized expression of a miRNA was zero in samples, this data was modified as 0.01; if the normalized expression of a miRNA in both compared samples was less than 1, the miRNA was not used in the analysis of differential expression. Next, Poisson distribution model for P-value calculation was used for estimating the statistical significance of miRNA expression changes under low N stress,and the formula shown below:

P-value formula:
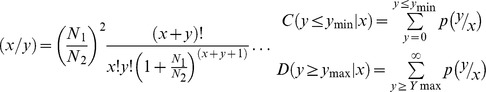



Finally, the miRNAs meeting the following criteria were considered as differentially expressed miRNAs: (i) p-value should be less than 0.05; (ii) fold change or log_2_ ratio of normalized counts between low-N stress and corresponding control library was greater than 1 or less than −1.

### Quantitative Real-time PCR Analysis

RT-qPCR is widely used to measure the gene expression variation, and the choice of suitable genes to use as reference genes is a crucial factor for interpretation of RT-qPCR results. The expression of reference genes is known to vary considerably under different experimental conditions, therefore, these reference genes should be evaluated for their stability of expression. In the present study, two protein-coding genes (TUA5, ACT) and three miRNAs (gma-miR1520d-3p, gma-miR156b-5p, gma-miR166a-5p) were selected to analyze their expression stability in all eight No. 116 libraries. All primers of the nine candidate reference genes are listed (Table S1). Primer sequences for the two mRNA housekeeping genes were chosen based on current literature [57], [58], and the stem-loop primers, used for miRNAs candidate reference genes, were designed according to Chen et al [59], which consisted of a self-looped 44 bp sequence (5′-GTCGTATCCAGTGCAGGGTCCGAGGTATT- CGCACTGGATACGAC-3′) and 6 variable nucleotides that were specific to the 3′ end of the miRNA sequence. RT-qPCR was performed as previously described [59], [60]. Briefly, the total RNA was reverse-transcribed using miRNA specific stem-loop primers or Oligo(dT) with M-MLV (Takara, China), then cDNA products were used as template for qPCR with gene-specific primers and universal reverse primer (5′GTGCAGGGTCCGAGGT-3′). The qPCR reactions were performed in triplicates on IQ™5 and MyiQ™ Real-Time PCR Detection Systems (Bio-Rad) using SYBR-Green. Each PCR reaction was performed in a final volume of 20 µl containing containing 10 ml 2×SYBR Premix Ex Taq II (TaKaRa, Japan), 0.25 mM of each primers and cDNA from reverse-transcribed from 100 pg total RNA using the following protocol: 95°C for 5 min, 40 cycles of 95°C for 10 sec, 60°C for 10 sec and 72 °C for 15 sec. At the end of the cycling protocol, a melting-curve analysis from 55 to 95°C was performed to determine specificity of the amplified products. All RT-qPCR reactions were performed with three biological replicates. RT-qPCR data was analyzed with geNorm software (V3.50) to determine the stability of candidate reference genes expression [61].

After determining the appropriate reference genes, 12 miRNAs were randomly selected for RT-qPCR assays to validate the reliability of Solexa/Illumina sequencing technology. These RT-qPCR assays were performed as described above. All the primers used are listed (Table S1). The relative expression levels of these miRNAs were calculated by delta-Ct method [62], which first transformed the Ct values of interest miRNA and reference genes to quantities using delta-Ct, then dividing the quantities of interest miRNA by the geometric mean of the reference genes. The mean and SD are calculated from the triplicate RT-qPCR assays. Student's t-test was used for statistical analysis of RT-qPCR data.

### Prediction of Potential Target Genes for Differentially Expressed miRNAs

Target prediction of differentially expressed miRNAs was performed based on methods described by Allen et al. [63]. Mature miRNA sequences were used as query to search against the *Glycine max* unigene database by the psRNATarget server using the following stringent parameters : (i) No more than two mismatches between miRNA and its target (G-U bases count as 0.5 mismatches), (ii) No mismatches in positions 10–11 of miRNA and its target duplex. The functional annotation of identified putative miRNA targets were inspected on the Phytozome using the Blast2Go (B2G) software suite v2.3.1 with the default parameters.

## Results

### An Overview of Small RNA Libraries Data Sets by High-throughput Sequencing

In our previous study, the exposure of No.116 (low N tolerant variety) and No.84-70 (low N-sensitive variety) to short-term (0.5 h, 2 h, 6 h, 12 h) and long-term (3 d, 6 d, 9 d, 12 d) low N stress resulted in different morphological and physiological changes [49]. Furthermore, 3231 differentially expressed genes involved in 22 metabolism and signal transduction pathways were identified through digital gene-expression [49]. In this study, to identify miRNAs in response to low N stress, sixteen small RNA libraries were constructed and sequenced using Illumina HiSeq 2000, yielding a total of 361,296,585 sRNA raw reads (more than twenty million for each library). After removing low quality reads, adapters, poly-A sequences and short RNA reads smaller than 18 nucleotides, 348,651,354 (96.50%) clean reads including 52,351,387 unique sequences were obtained from all these libraries. For clean reads, 116RS library produced the least clean reads (19,583,940) and 116RL library yielded the most clean reads (23,859,206), while for unique sequences, 84SLC library generated the least unique sequences (955,962) and 116RL library showed the most abundant unique sequences (4,853,221). Although the average sequenced frequency of a unique sequence was from 4.8 (116RLC library) to 20.7 (84SLC library), over 74% of the unique sequences were only sequenced once in these libraries, indicating that sequencing was far from saturated (data not shown). These unique sequences were then perfectly mapped to the soybean genome using SOAP2 software, and the results showed that over 57.4% of unique sequences matched the soybean genome in these libraries. Among these libraries, the highest proportion of unique sequences mapped to the soybean genome came from the 84SS library (78.26%) and the lowest proportion come from the 116RLC library (57.45%) (Table 1).

**Table 1:** Statistics of sequenced reads from all libraries.

libraries	raw reads	clean reads	Unique reads	Total RNAs mappedto genome	Unique RNAs mapped to genome
116RS	20,563,913	19,583,940	3,740,997	15650469(79.91%)	2259049 (60.39%)
116RSC	21,657,642	20,229,978	3,807,854	16533150(81.73%)	2364006(62.08%)
116RL	24,982,431	23,859,206	4,853,221	18638795(78.12%)	2837312(58.46%)
116RLC	21,671,329	20,420,678	4,211,257	15594096(76.36%)	2419218(57.45%)
84RS	23,001,960	21,529,981	3,909,940	17589661(81.70%)	2619015(66.98%)
84RSC	21,440,496	20,361,472	3,942,964	16141497(79.27%)	2449851(62.13%)
84RL	23,201,153	22,194,791	4,444,930	17545304(79.05%)	2746226(61.78%)
84RLC	25,102,491	23,835,520	4,146,988	19254010(80.78%)	2510625(60.54%)
116SS	23,151,683	22,694,642	3,409,006	19450015(85.70%)	2558524(75.05%)
116SSC	23,103,654	22,623,753	3,748,081	18926241(83.66%)	2760503(73.65%)
116SL	22,275,675	21,991,532	1,342,837	19965183(90.79%)	984532(73.32%)
116SLC	21,155,459	20,944,820	1,332,422	19331120(92.30%)	987783(74.13%)
84SS	24,004,607	23,602,217	3,294,804	20396440(86.42%)	2578600(78.26%)
84SSC	24,158,394	23,595,539	3,632,865	20461997(86.72%)	2809572(77.34%)
84SL	21,663,173	21,316,261	1,577,259	19181538(89.99%)	1188381(75.34%)
84SLC	20,162,525	19,867,024	955,962	17949389(90.35%)	708189(74.08%)
Total	361,296,585	348,651,354	52,351,387	292,608,905	34,781,386

Clean reads are those remaining after low-quality reads have been removed from total raw reads. Unique reads are different types of clean reads. The number of the total clean reads and unique reads from the sixteen libraries that matched to the genome sequences are also listed.

Note: 116RS, 116-root short-term treatment; 116RL, 116-root long-term treatment; 84RS, 84-70-root short-term treatment; 84RL, 84-70-root long-term treatment; 116SS, 116-shoot short-term treatment; 116SL, 116-shoot long-term treatment; 84SS, 84-70-shoot short-term treatment; 84SL, 84-70-shoot long-term treatment; 116RSC, 116-root short-term control; 116RLC, 116-root long-term control; 84RSC, 84-70-root short-term control; 84RLC, 84-70-root long-term control;116SSC, 116-shoot short-term control; 116SLC, 116-shoot long-term control; 84SSC, 84-70-shoot short-term control; 84SLC, 84-70-shoot long-term control.

**Figure 1:**
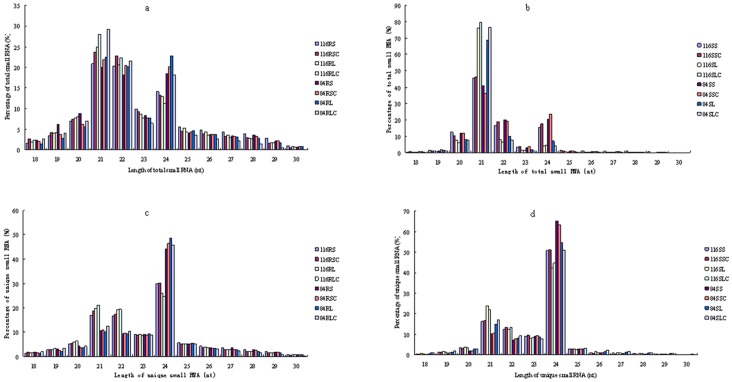
The length distribution of soybean sRNAs. (a) Length distribution of total sRNAs reads in eight root libraries. The y-axis indicates percent of total sRNAs, and the x-axis indicates length of total sRNAs (nt). (b) Length distribution of total sRNAs reads in eight shoot libraries: The y-axis indicates percent of total sRNAs, and the x-axis indicates length of total sRNAs (nt). (c) Length distribution of unique sRNAs reads in eight root libraries. The y-axis indicates percent of unique sRNAs, and the x-axis indicates length of unique sRNAs(nt). (d) Length distribution of unique sRNAs reads in eight shoot libraries. The y-axis indicates percent of unique sRNAs, and the x-axis indicates length of unique sRNAs(nt). 116RS, 116-root short-term treatment; 116RL, 116-root long-term treatment; 84RS, 84-70-root short-term treatment; 84RL, 84-70-root long-term treatment; 116SS, 116-shoot short-term treatment; 116SL, 116-shoot long-term treatment; 84SS, 84-70-shoot short-term treatment; 84SL, 84-70-shoot long-term treatment; 116RSC, 116-root short-term control; 116RLC, 116-root long-term control; 84RSC, 84-70-root short-term control; 84RLC, 84-70-root long-term control; 116SSC, 116-shoot short-term control; 116SLC, 116-shoot long-term control; 84SSC, 84-70-shoot short-term control; 84SLC, 84-70-shoot long-term control.

Size distribution of sRNAs based on both total sRNAs reads and unique sRNAs reads were analyzed (Figure 1).The majority of the total sRNAs reads were found to be in the range of 21 nt to 24 nt in length in all 16 libraries. Three major peaks at 21 nt, 22 nt and 24 nt were observed in eight root libraries (Figure 1a), while 21-nt small RNAs in total sRNA reads were dominant in eight shoot libraries (Figure 1b). The length distribution of unique sRNA reads revealed that the 24 nt sRNAs were the most abundant class in all libraries and it was followed by 21, 22 nt sRNAs (Figure 1c and Figure 1d). Overall, although these small RNAs were unevenly distributed according to their length in all libraries, a proportion of small RNAs of a certain length was found to be similar among all libraries. To further compare the average abundance of sRNAs with different lengths, the ratio of raw and unique sequences was calculated, which found that the ratio of sRNAs varied along with length, and the 21 nt sRNAs showed the highest redundancy. The result was consistent with previous reports from other plant species [36], [64]–[65].

### The Most Abundant miRNA Variants Corresponded to Soybean known miRNAs

In the process of detecting known miRNAs in soybean, we found that in some cases, the most abundant sequences among all unique sequences mapped to the known pre-miRNAs of soybean were not annotated miRNA sequences in miRBase. Thus, we turned to search for the most abundant variants and compared them among all 16 libraries to determine if these variants were more abundant than annotated miRNA sequences in most libraries. In sixteen libraries, a total number of 349 known pre-miRNAs belonging to 158 miRNA families were analyzed to determine the most abundant variants by investigating the distribution of unique RNA sequences on known pre-miRNAs. In some libraries,some of the known pre-miRNAs could not be well-supported by unique RNA sequences, therefore, numbers of known pre-miRNAs analyzed were different in every library, and number of pre-miRNAs (348) analyzed in 84RLC library was the most (Table 2 and Table S2).

**Table 2:** Summary of unified miRNA variants mapping to known miRNAs precursors.

	116RS	116RSC	116RL	116RLC	84RS	84RSC	84RL	84RLC	116SS	116SSC	116SL	116SLC	84SS	84SSC	84SL	84SLC	Total
precursor (location)	342	346	345	345	343	347	346	348	345	344	328	330	343	343	335	333	349
families	153	156	155	154	153	156	156	157	157	157	150	146	157	157	155	151	158
miRNA variants	354	358	358	355	356	360	359	361	355	354	338	340	352	353	343	342	362
miRNA*	181	193	204	201	206	199	205	206	195	206	157	164	189	195	160	151	267

Precursors are annotated known miRNA precursors (pre-miRNAs) in miRBase. Families are annotated known miRNA families in miRBase. miRNA variants are the most abundant sequences mapping to 5′ arm or 3′ arm of known miRNAs precursors. miRNA* are the sequences which can form miRNA::miRNA* duplexes with miRNA variants.

When the most abundant variants located on their corresponding pre-miRNA 5′ arm or 3′ arm were searched and compared in all 16 libraries, we found that although some of the most abundant variants were same in all 16 libraries, some of the most abundant variants did not coincide among 16 libraries. In order to further study the differential expression analysis, these most abundant variants that showed differences among 16 libraries needed be unified. Therefore, we followed the rule that if a unique RNA sequence mapped to its corresponding pre-miRNA 5′ arm or 3′ arm was the most abundant variant in a majority of these libraries, moreover, and if the sequenced frequency of other unique RNA sequences (most abundant variant in the other libraries) was close to frequency of the former unique RNA sequence in the same libraries, the unique RNA sequence that was most abundant variant in majority of these libraries was assumed as the “unified most abundant variant” in all 16 libraries. Otherwise, other unique RNA sequences that was most abundant variant in the other libraries could not be replaced by the unique RNA sequence that was the most abundant variant in a majority of these libraries to calculate sequenced reads. For example, for gma-miR151-3p, gma-miR166h-3p, gma-miR172i-3p, gma-miR1510a-5p and gma-miR398b-5p, their most abundant variants on their corresponding precursors in 16 libraries were different with greatly varied sequenced reads in the same libraries and couldn’t be unified to a sole sequence (Table S2, Figure S1). Interestingly, for gma-miR4361-3p and gma-miR4368b-3p, their most abundant variants on respective miRNA precursors were almost the same in 8 libraries from the same variety, however, they were different between libraries from the two varieties (Table S2, Figure S1). This implied that the most abundant variants might be variety –specific.

In classical plant miRNA biogenesis pathway, a pre-miRNA is cleaved into miRNA::miRNA*duplex, the miRNA of which joins with Argonaute (AGO) to form the RNA-induced silencing complex (RISC) to regulate gene expression. In most cases, miRNA*is quickly degraded, so it is found at a much lower frequency than miRNAs [66]. We assumed the most abundant variant on the whole stem of a pre-miRNA (5′ arm or 3′ arm) as miRNA variant, while the most abundant variant on the opposite arm of miRNA variant as miRNA* variant. The analysis of the most abundant variant showed that miRNA variants for over 50% of known pre-miRNAs were the same in these 16 libraries, while the miRNA* variants were less consistent (Table S2,Figure S1). For example, their corresponding most abundant variants of gma-miR167g, gma-miR169f and gma-miR394b were found to be located in their pre-miRNAs 5′ arm (as miRNA variants) and were consistent in 16 libraries. They also had more sequence reads than the corresponding miRNA* variants located in the pre-miRNAs 3′ arm, and the miRNA* variants were different in these 16 libraries. Certainly, miRNA variants could come from their pre-miRNAs 3′ arm and were slightly more prevalent than miRNA variants from the pre-miRNAs 5′ arm (Table S2). In addition, we found that for some miRNAs, such as gma-miR169l, gma-miR171b and gma-miR4414, their corresponding miRNA variants in some libraries were located on the pre-miRNAs 5′ arm, while in other libraries located on the pre-miRNAs 3′ arm (Table S2, Figure S1). These results revealed the alternative use of the pre-miRNAs 5′ and pre-miRNAs 3′ arms as well as the complexity of the mature miRNA variants generating processes in different libraries.

For analysis of these unified miRNA variants’ sequenced reads, these unique RNA sequences located between +2 and −2 nt away from unified miRNA variants on their corresponding pre-miRNAs were included in our calculations. We found that gma-miR3522 5p was the most abundantly expressed and was sequenced 79410810 in 16 libraries, followed by some species of gma-miR1507a/b-3p, gma-miR156d/g/i/j/l/m-5p and gma-miR166u-3p. We also calculated sequenced reads of miRNA* variants, and the miRNA* variants were considered to be identified even if miRNA* variants were sequenced once in one library of 16 libraries, thus a total of 267 miRNA* variants were identified in 16 libraries (Table 2 and Table S2).

Previous studies showed that a single miRNA precursor could produce two or more distinct miRNAs [53]. In this study, we also found that some pre-miRNAs, such as pre-miR159a, pre-miR319a, pre-miR394a, and pre-miR2118a could generate distinct abundant sequences on their 5′ arm or 3′ arms, the positions of which didn’t overlap and the sequence frequencies were high enough. Most importantly, we found most of their corresponding miRNA*s sequences, indicating they were likely to be the products of DCL1 processing. These distinct abundant sequences from the same precursor could be annotated as different miRNA variants of the same precursor, amplifying analyzed known miRNA variants to 362 in 16 libraries (Table 2, Table S2, Figure S1).

**Figure 2:**
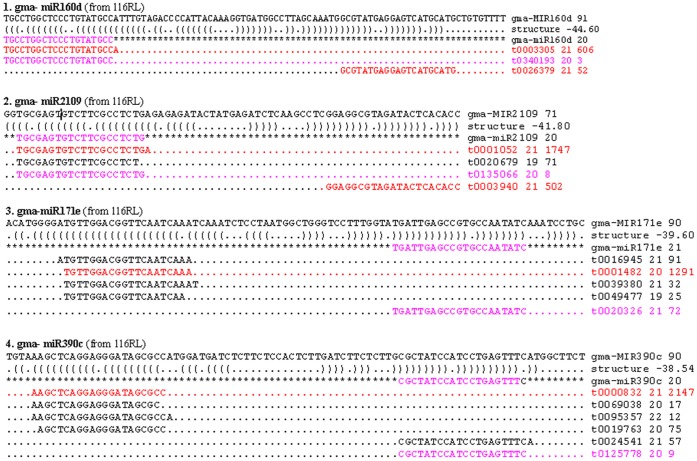
Examples where the most abundant sequences were different from the annotated miRNA. only sequences with more than 10 reads are shown except the most abundant variants and the annotated miRNA, the sequences that was the most abundant variants on the two arm of pre-miRNAs were shown in red, and the annotated miRNAs were shown in pink.

To further characterize these miRNA variants, we compared them with annotated mature soybean miRNAs in miRBase (Table S2, Figure S1, Figure 2). Though many annotated miRNAs were the most abundant sequences on their corresponding pre-miRNAs in all libraries, almost 50% of the miRNA variants were not consistent with annotated miRNAs in miRBase. For example, for gma-miR160d, gma-miR2109, and gma-miR482b, although the most abundant variants on both arms of respective pre-miRNAs were different with annotated miRNAs, they were the same in these 16 libraries and could form miRNA::miRNA* duplex, supporting that they were likely authentic miRNAs or miRNA*s and could substitute for the annotated miRNAs. In addition, we found that some miRNA variants were located on the opposite arms of the annotated miRNAs on their corresponding pre-miRNAs. For example, for gma-miR171e, gma-miR159d and gma-miR390c, their corresponding miRNA variants were located on the corresponding pre-miRNAs 5′ arms, whereas their annotated miRNA were located on the corresponding pre-miRNAs 3′ arms.

### Putative Novel miRNAs in Soybean

Besides the unique RNA sequences aligned known miRNA precursors, there were still numerous unclassified small RNAs in these 16 libraries, some of which might be novel miRNAs. The Mireap software was used to predict novel miRNAs with adjusted parameters, which were suitable for plant miRNAs identification [67]. Approximately, 3000 loci were predicted with miRNA precursor-like stem-loop structures in the 16 libraries. Since the most abundant sequences on these new loci weren’t always consistent among the libraries, we adopted the above rule of known miRNA variants to unify them to count sequenced frequency. To improve accuracy of a new miRNA prediction, we adopted the following critical criterion: (1) miRNA* was detected in at least one library, (2) total sequenced frequency of a novel miRNA in 16 libraries were over 100, as the use of low expression miRNAs is prone to high false positive. Thus only 90 loci belonging to 55 miRNA families were annotated as new miRNAs, and each library had varied novel miRNAs (Table 3 and Table S3). Generally, these new miRNAs were named temporarily in the form of novel-soy-number-3p/5p,. For some paralogous loci of newly identified miRNAs that could be classified into the same families, they were designated as novel-soy-number-letters-3p/5p. For example, novel-soy0055 have nine paralogous loci that were named novel-soy0055a∼novel-soy0055i, respectively (Table S3). According to the sequencing reads, novel-soy0001-3p was found to be the most abundantly expressed novel miRNA, followed by novel-soy0045-5p, novel-soy0055i-3p and novel-soy0027-3p (Table S3).

**Table 3:** Summary of new miRNAs.

	116RS	116RSC	116RL	116RLC	84RS	84RSC	84RL	84RLC	116SS	116SSC	116SL	116SLC	84SS	84SSC	84SL	84SLC	Total
novel miRNA	89	89	89	89	87	89	88	88	89	89	85	88	80	88	81	84	90
miRNA*	29	31	38	41	25	33	28	24	32	47	22	17	23	36	21	18	74
families	54	54	54	54	52	54	53	53	54	54	52	53	50	53	51	51	55

Novel miRNAs are the small sequences which meet the critical criterion of novel miRNA annotation. miRNA* are the sequences which can form miRNA::miRNA* duplexes with novel miRNA. Families are the novel miRNAs with similar sequences.

In accordance with the known miRNAs, these newly identified miRNAs derived from predicted hairpin structures ranged from 80 to 376 nt, and the minimum folding free energy (MFE) for these structures hairpin structures was found to be less than 20 kcal mol^−1^. Almost 50% of these newly identified miRNAs were 21 nt miRNAs beginning with a U, and the pre-miRNAs 5′ and pre-miRNAs 3′ arms were alternatively used as sources of miRNA in different libraries (Table S3).

To confirm whether these new miRNAs were homologous with known miRNAs, we compared them with the known miRNAs from all plant species deposited in miRBase. Our results showed that eight new miRNAs or miRNAs*(novel-soy0001, novel-soy0006, novel-soy0013, novel-soy0020, novel-soy0025, novel-soy0026, novel-soy0044 and novel-soy0045) were orthologues of known miRNAs identified in different plant species (Table S3). In addition, although most new miRNAs were independently transcribed from intergenic regions of the genome, we found that a few new miRNAs loci were located in the introns or exons of the protein-coding genes. The positions of these new miRNAs on protein-coding genes as well as the functions of protein-coding genes have been summarized (Table 4). These genes are involved in distinct plant physiological processes except that the functions of some genes were unclear.

**Table 4:** Some new miRNAs located in the introns or exons of protein-coding genes.

New miRNA name	Genomic_locus	Position of new miRNA located on corresponding gene	gene function
novel-soy0014	Gm19∶36915900∶36915999:+	Glyma19g29360.1_intr10	Predicted ATPase (PP-loop superfamily)
novel-soy0017	Gm10∶50870523∶50870801:−	Glyma10g44540.1_intr6	Starch binding domain
novel-soy0018	Gm13∶41358349∶41358450:+	Glyma13g40930.2_intr2	RRM motif-containing protein
novel-soy0027	Gm13∶35514890∶35515064:+	Glyma13g33830.1_intr3	Hydroxyindole-O-methyltransferase and related SAM-dependent methyltransferases
novel-soy0028	Gm02∶42141386∶42141490:+	Glyma02g36710.1_intr3	translation initiation factor IF-3
novel-soy0029	Gm03∶45559347∶45559504:−	Glyma03g39500.1_intr2	26S proteasome regulatory complex, ATPase RPT5
novel-soy0030	Gm08∶41436001∶41436101:−	Glyma08g41460.2_intr2	serine–glyoxylate transaminase
novel-soy0031	Gm08∶4270886∶4271063:−	Glyma08g06010.1_intr8	Thioredoxin
novel-soy0033	Gm18∶13659100∶13659200:+	Glyma18g14150.1_intr3	unknown
novel-soy0034	Gm10∶48411098∶48411434:−	Glyma10g41290.1_intr1	Predicted signal transduction protein,PCD6 interacting protein-related
novel-soy0035	Gm14∶26871028∶26871361:+	Glyma14g22750.1_intr3	unknown
novel-soy0036	Gm08∶46321055∶46321233:−	Glyma08g47470.2_intr3	Activator of Hsp90 ATPase
novel-soy0037	Gm14∶33572225∶33572417:−	Glyma14g27380.1_intr9	unknown
novel-soy0038	Gm17∶14170511∶14170612:−	Glyma17g17360.1_intr1	NADH:ubiquinone oxidoreductase, NDUFS4/18 kDa subunit
novel-soy0039	Gm20∶2071915∶2072036:+	Glyma20g02450.1_intr1	unknown
novel-soy0040	Gm11∶33167338∶33167491:+	Glyma11g31810.1_intr4	Hsp70-interacting protein
novel-soy0041	Gm16∶29006145∶29006227:+	Glyma16g25080.1_intr1	Leucine Rich Repeat PROTEIN
novel-soy0042	Gm06∶44529277∶44529430:−	Glyma06g41240.1_exon3	Apoptotic ATPase,Leucine Rich Repeat
novel-soy0045	Gm18∶61442586∶61442692:−	Glyma18g53060.1_exon1	unknown
novel-soy0050a	Gm20∶39792640∶39792742:−	Glyma20g31140.3_intr7	unknown
novel-soy0050b	Gm10∶893455∶893562:+	Glyma10g01210.1_intr2	SBP domain
novel-soy0050c	Gm11∶36844937∶36845041:−	Glyma11g35100.1_intr5	Arginyl-tRNA-protein transferase
novel-soy0050d	Gm13∶37718475∶37718554:+	Glyma13g36430.1_intr2	Ypt/Rab-specific GTPase-activating protein GYP7 and related proteins
novel-soy0051a	Gm13∶38887195∶38887345:+	Glyma13g37960.2_intr1	Universal stress protein family
novel-soy0051b	Gm09∶109565∶109722:−	Glyma09g00360.1_intr2	RhoGAP domain
novel-soy0052a	Gm05∶40112962∶40113078:+	Glyma05g36260.1_intr14	Protease family M24(methionyl aminopeptidase,aminopeptidase P)
novel-soy0052b	Gm05∶15006208∶15006324:−	Glyma05g14210.1_intr8	DNA double-strand break repair RAD50 ATPase
novel-soy0053a	Gm03∶44205908∶44206007:+	Glyma03g37670.1_intr3	DNA-directed RNA polymerase subunit E
novel-soy0053b	Gm19∶46707950∶46708048:+	Glyma19g40280.2_intr3	DNA-directed RNA polymerase subunit E
novel-soy0053c	Gm19∶5268050∶5268406:+	Glyma19g05020.1_intr5	ATP sulfurylase (sulfate adenylyltransferase)
novel-soy0053d	Gm20∶44946295∶44946393:−	Glyma20g36970.1_intr24	Myosin class V heavy chain
novel-soy0053e	Gm08∶34672874∶34672969:−	Glyma08g36580.1_intr5	unknown
novel-soy0053f	Gm05∶29437141∶29437234:−	Glyma05g23710.2_intr4	LSD1 zinc finger
novel-soy0053g	Gm20∶818958∶819056:−	Glyma20g01180.2_intr12	acetyl-CoA acyltransferase
novel-soy0053h	Gm02∶5732006∶5732122:+	Glyma02g07190.1_intr1	DHHC-type Zn-finger proteins
novel-soy0053i	Gm19∶45160636∶45160733:+	Glyma19g38180.1_intr2	Predicted alpha/beta hydrolase
novel-soy0053j	Gm08∶5615228∶5615326:−	Glyma08g07840.1_intr1	COPII vesicle protein

### The miRNAs Responsive to Short-term and Long-term N Limitation in Soybean Roots

**Figure 3:**
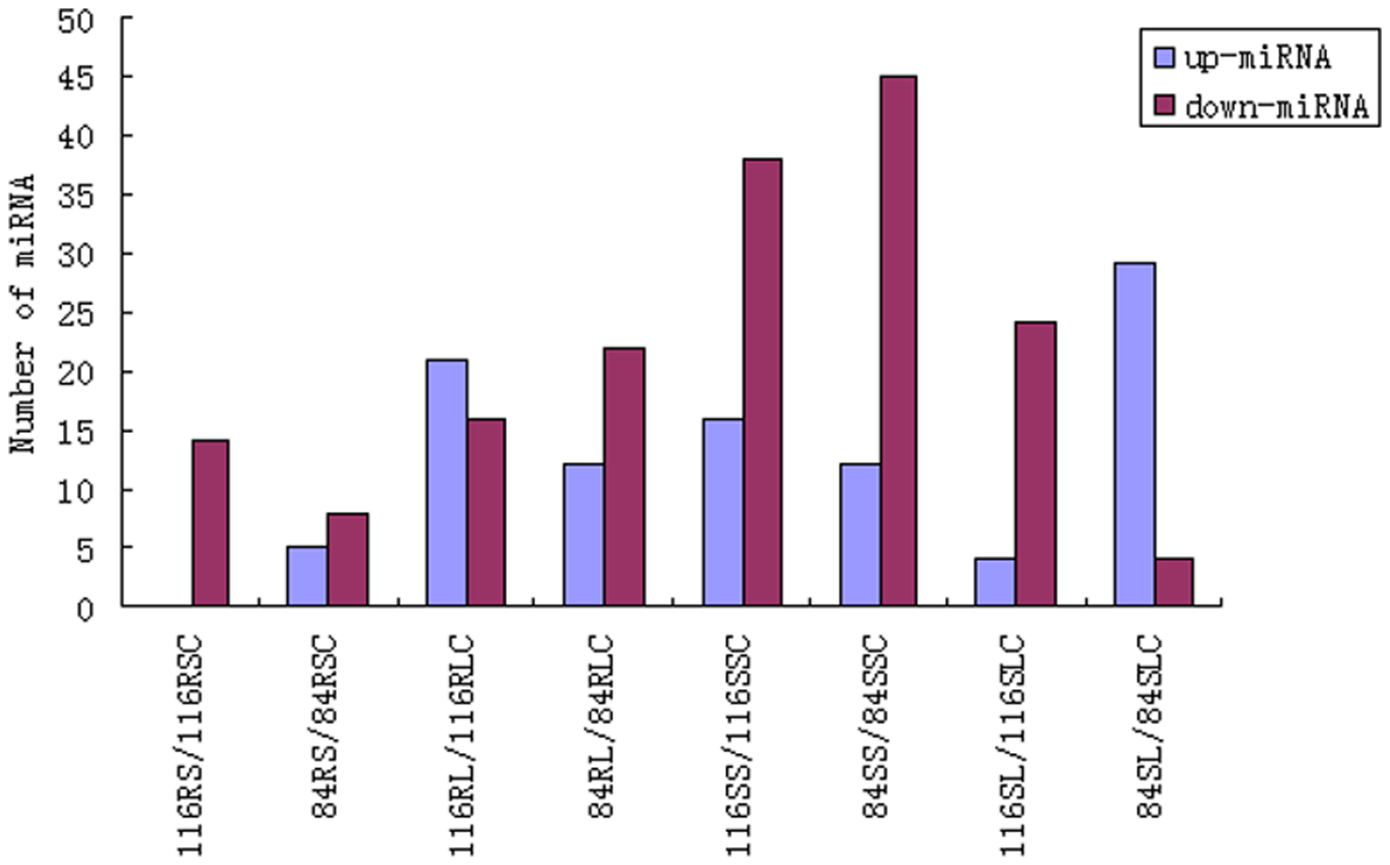
Numbers of up-regulated and downregulated miRNA were summarized in compared libraries. 116RS, 116-root short-term treatment; 116RL, 116-root long-term treatment; 84RS, 84-70-root short-term treatment; 84RL, 84-70-root long-term treatment; 116SS, 116-shoot short-term treatment; 116SL, 116-shoot long-term treatment; 84SS, 84-70-shoot short-term treatment; 84SL, 84-70-shoot long-term treatment; 116RSC, 116-root short-term control; 116RLC, 116-root long-term control; 84RSC, 84-70-root short-term control; 84RLC, 84-70-root long-term control;116SSC, 116-shoot short-term control; 116SLC, 116-shoot long-term control; 84SSC, 84-70-shoot short-term control; 84SLC, g00484-70-shoot long-term control.

**Figure 4:**
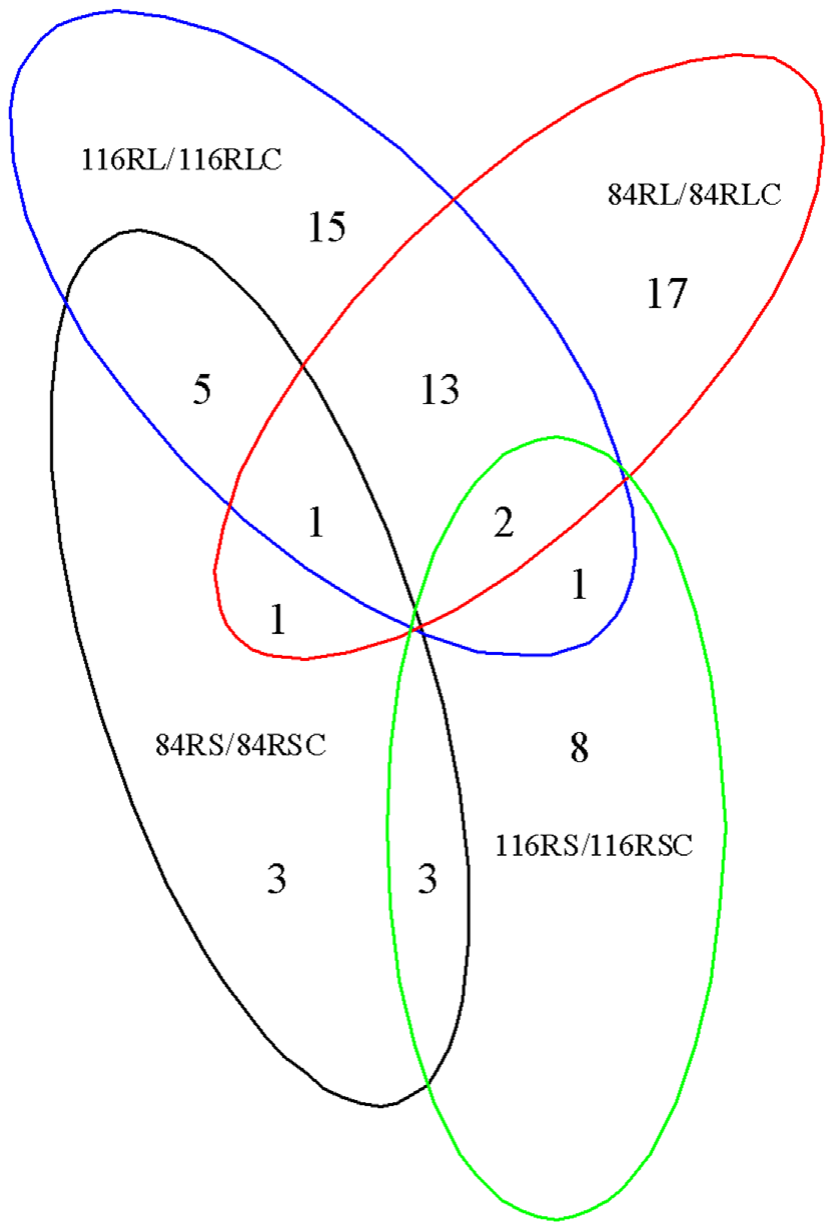
Specific and common response miRNAs in soybean roots from differential compared libraries. 116RS, 116-root short-term treatment; 116RL, 116-root long-term treatment; 84RS, 84-70-root short-term treatment; 84RL, 84-70-root long-term treatment; 116-root short-term control; 116RLC, 116-root long-term control; 84RSC, 84-70-root short-term control; 84RLC, 84-70-root long-term control.

To identify low N-responsive miRNAs (known miRNAs and new miRNAs) in soybean roots, we compared miRNA expression profiling between short-term or long-term low N stress and corresponding control libraries in both genotypes. Specifically, the sequenced amount of a specific miRNA was first normalized as transcripts per million (TPM), then the log_2_ ratios between low N stress and corresponding control libraries and P-value based on Poisson distribution model were calculated. To minimize noise and improve accuracy, we only selected the miRNAs with sequence reads over 100 in at least one library for comparison. P-value ≤0.05 and the absolute value of log_2_ ratio ≥1.0 as a threshold were used to judge the statistical significance of miRNA expression. The miRNAs with log_2_ ratio ≥1.0 were designated as ‘up-regulated’, while the miRNAs with log_2_ ratio ≤−1.0 as ‘down-regulated’ (Figure 3, Figure 4 and Table S4). Results showed that in No.116 variety (tolerant genotype), 14 known miRNAs belonging to 5 miRNA families were found to be significantly differentially expressed in response to short-term low N stress, and these 14 miRNAs were all down-regulated and reduction in gma-miR2109-3p expression was the highest. In No.84-70 variety (sensitive genotype), 13 known miRNAs belonging to 8 miRNA families were identified to be significantly differentially expressed. Among 13 known miRNAs, 5 known miRNAs (i.e. gma-miR1510a-5p,gma-miR396b/d/g-3p) were up-regulated while the other known miRNAs (i.e. gma-miR1512a-5p, gma-miR5372-5p) were down-regulated. Among the significantly differentially expressed miRNAs responsive to short-term N limitation from both genotypes, 3 known miRNAs (gma-miR408a/b/c-5p) were found to be common and down-regulated. Under long-term low N stress condition, 36 known miRNAs belonging to 12 miRNA families as well as one novel miRNA (novel-soy0006-3p) were identified to be significantly differentially expressed in No.116 variety. Among these 36 miRNAs, 15 known miRNAs (i.e.gma-miR396b/d/g-3p, gma-miR482a/c-3p, gma-miR2109-3p) and the novel miRNA were found to be down-regulated while the other known miRNAs were up-regulated. In No.84-70 variety, 34 known miRNAs belonging to 15 miRNA families were identified to be significantly differentially expressed. Among 34 known miRNAs, 12 known miRNAs (i.e. gma-miR1512b -5p, gma-miR171c/e-5p, gma-miR482a-5p) of which were up-regulated while the other known miRNAs (i.e. gma-miR156b/f-5p, gma-miR169c/p/s-5p) were down-regulated. Among these significantly differentially expressed miRNAs responsive to long-term N limitation from both genotypes, 16 known miRNAs in 7 miRNA families (i.e.gma-miR1512c-5p, gma-miR159a-3p-1, gma-miR159b/f-5p-2, gma-miR169c/e/h/p/s-5p, and gma-miR862a/b -5p) were common. To identify the common significantly differentially expressed miRNAs under both short-term and long-term low N stress conditions, miRNAs significantly differentially expressed in response to short-term or long-term low N stress were further compared. The results showed that in No.116 variety roots, 3 significantly differentially expressed known miRNAs belonging to 2 miRNA families(gma-miR159a/e-3p-1, gma-miR2109-3p) were common under short-term and long-term low N stress conditions, while in No.84-70 variety roots, 2 known miRNAs belonging to 2 miRNA families (gma-miR1510a-5p, gma-miR5559-5p) were common under both short-term and long-term low N stress conditions. Among both short-term and long-term low N-responsive miRNAs from roots of both genotypes, no significantly differentially expressed miRNA was found to be common.

### The miRNAs Responsive to Short-term and Long-term N Limitation in Soybean Shoots

**Figure 5:**
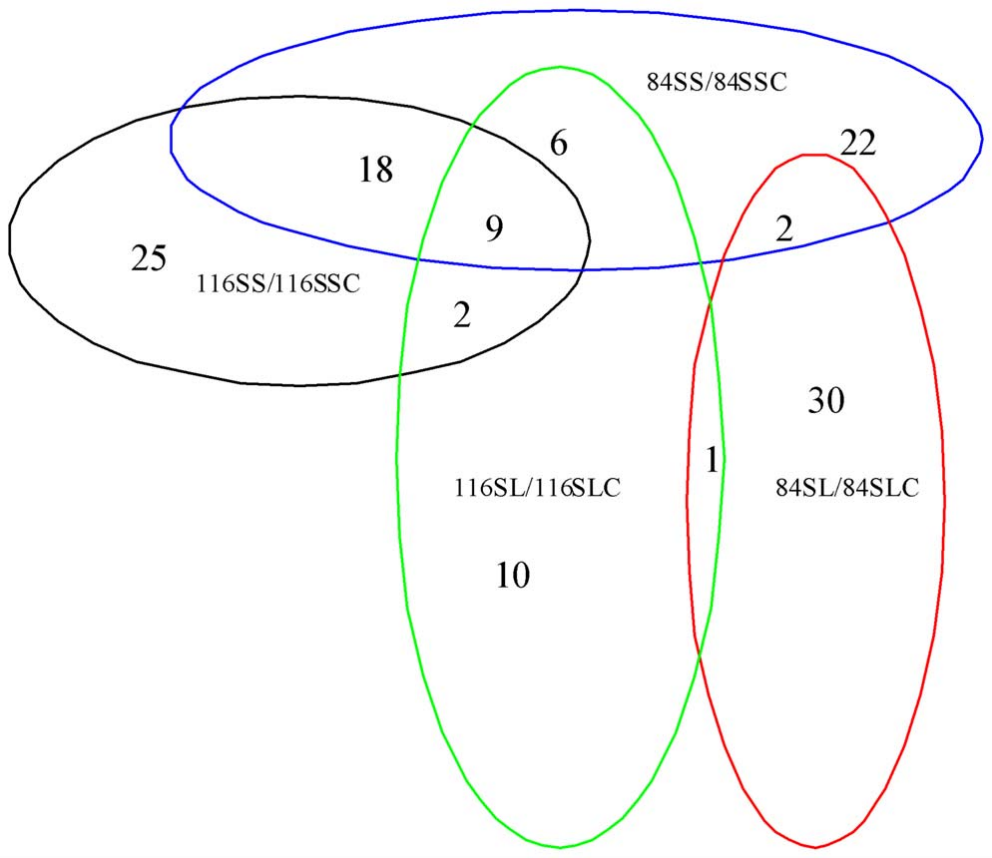
Specific and common response miRNAs in soybean shoots from differential compared libraries. 116SS, 116-shoot short-term treatment; 116SL, 116-shoot long-term treatment; 84SS, 84-70-shoot short-term treatment; 84SL, 84-70-shoot long-term treatment; 116SSC, 116-shoot short-term control; 116SLC, 116-shoot long-term control; 84SSC, 84-70-shoot short-term control; 84SLC, 84-70-shoot long-term control.

In soybean shoots, miRNAs responsive to short-term or long-term low N stress were investigated using the above mentioned method (Figure 3, Figure 5 and Table S4). Results displayed that in No.116 variety, 54 known miRNAs belonging to 19 miRNA families were identified to be significantly differentially expressed in response to short-term low N stress, and 16 of these known miRNAs belonging to 8 miRNA families (i.e. gma-miR166i-5p, gma-miR396b/d/g-3p, gma-miR408a/b/c/d-3p) were found to be up-regulated while the other known miRNAs (i.e. gma-miR160a/b/c/d/e-5p,gma-miR171c-5p,gma-miR398c-5p) were down-regulated. In No.84-70 variety, 56 known miRNAs belonging to 15 miRNA families as well as one novel miRNA (novel-soy0043-5p) were found significantly differentially expressed, and among them 12 known miRNAs belonging to 6 miRNA families (i.e.gma-miR159a/e-3p-1, gma-miR2119-3p, gma-miR482b/d-3p) were up-regulated while the other known miRNAs (i.e. gma-miR156b/f-5p, gma-miR171b/e/h-5p, gma-miR390a/b/c/d-5p) and the novel miRNA were down-regulated. Among these miRNAs responsive to short-term low N stress from shoot of both genotypes, 27 known miRNAs belonging to 7 miRNA families (i.e.gma-miR160/b/c/d/e-5p, gma-miR396b/c/d/f/g-5p, gma-miR2109-5p, gma-miR5372-5p, gma-miR394b/c-5p) were common. Under long-term low N stress condition, 28 known miRNAs belonging to 14 miRNA families were found significantly differentially expressed in No.116 variety, and among them 4 known miRNAs belonging to 2 miRNA families(gma-miR156p/t-5p, gma-miR156r-3p ma-miR5774b-5p) were found to be up-regulated while the other known miRNAs(i.e. gma-miR397a/b-5p, gma-miR398c-5p, gma-miR408a/b/c/d-5p) were down-regulated. In No.84-70 variety, 33 known miRNAs belonging to 15 miRNA families were significantly differentially expressed, of which 4 known miRNAs (gma-miR2119-3p, gma-miR398a/b-5p, gma-miR5786-5p) were down-regulated while other known miRNAs belonging to 15 miRNA families (i.e. gma-miR1507a/b/c-5p, ma-miR171e-5p, gma-miR168a/b-5p, gma-miR482a/c-3p) were up-regulated. Among these miRNAs responsive to short-term low N stress from shoot of both genotypes, only one known miRNAs(gma-miR5774b-5p) were found to be common. The miRNAs that were significantly differentially expressed in response to short-term and long-term low N stress were further compared, and the results showed that in No.116 variety, 11 known miRNAs belonging to 6 miRNA families (i.e.gma-miR160/b/c/d/e-5p, gma-miR398c-5p, gma-miR2109-3p) were significantly differentially expressed under both short-term and long-term low N stress conditions, while in No.84-70 variety, 2 known miRNAs belonging to 2 miRNA families (gma-miR171e-5p, gma-miR2119-3p) were both short-term and long-term low N-responsive miRNAs. Among both short-term and long-term low N-responsive miRNAs from shoots of both genotypes, no significantly differentially expressed miRNA was found to be common.

### Comparison of the Soybean Shoots miRNAs with Roots miRNAs Responsive to Short-term and Long-term N Limitation

The miRNAs that were found significantly differentially expressed in response to short-term or long-term low N stress in soybean shoots were further compared with significantly differentially expressed miRNAs in soybean roots (Table S4). The results showed that in No.116 variety, 9 known miRNAs belonging to 3 miRNA families (gma-miR159a/e-3p-1, gma-miR160/b/c/d/e-5p, gma-miR2109-3p, gma-miR2109-5p) were significantly differentially expressed in response to short-term low N stress in both shoots and roots of No.116 variety. Under long-term low N stress condition, 5 known miRNAs belonging to 4 miRNA families(gma-miR159a/e-3p-1, gma-miR2109-3p, gma-miR397b-3p, gma-miR408d-5p) were significantly differentially expressed in both shoots and roots of 116 variety. The significantly differentially expressed miRNAs in both shoots and roots of No.116 variety responsive to short-term were further compared with those responsive to long-term low N stress. The results showed that 3 known miRNAs in 2 miRNA families (gma-miR159a/e-3p-1, gma-miR2109-3p,) were common. In No.84-70 variety, 2 known miRNAs from 2 miRNA families (gma-miR5372-5p, gma-miR5559-5p) were found significantly differentially expressed in response to short-term low N stress in both shoots and roots of No.84-70 variety, while under long-term low N stress condition, 4 known miRNAs belonging to 3 miRNA families (gma-miR1510a/b-5p, gma-miR171e-5p, gma-miR482a-5p) were significantly differentially expressed in both shoots and roots of No.84-70 variety. The significantly differentially expressed miRNAs in both shoots and roots of No.84-70 variety responsive to short-term were further compared with those responsive to long-term low N stress. The results showed that no known miRNA was common.

### Confirmation of miRNAs by qRT-PCR

It is essential to evaluate the stability of candidate reference genes expression before RT-qPCR is used to determine the differential expression of genes. Two protein-coding genes (TUA5, ACT) and three miRNAs (gma-miR1520d-3p, gma-miR156b-5p, gma-miR166a-5p ) were selected for the analysis of their expression stability in all eight No. 116 libraries by geNorm software. The results showed that the average expression stability values (M) of miR156b-5p and miR1520d-3p were lower than those of the other genes in eight No. 116 libraries analyzed, which was consistent with previous reports [68]. To further determine the optimal number of reference genes for normalization, we calculated the pair-wise variation of these candidate reference genes. The combination of the two most stable genes (gma-miR1520d-3p, miR156b) was found to be sufficient for normalization purposes because the V2/3 value was lower than 0.15. Thus, the two miRNA genes (gma-miR1520d-3p, gma-miR156b-5p) were selected to normalize the level of gene expression(Figure S2).

**Figure 6:**
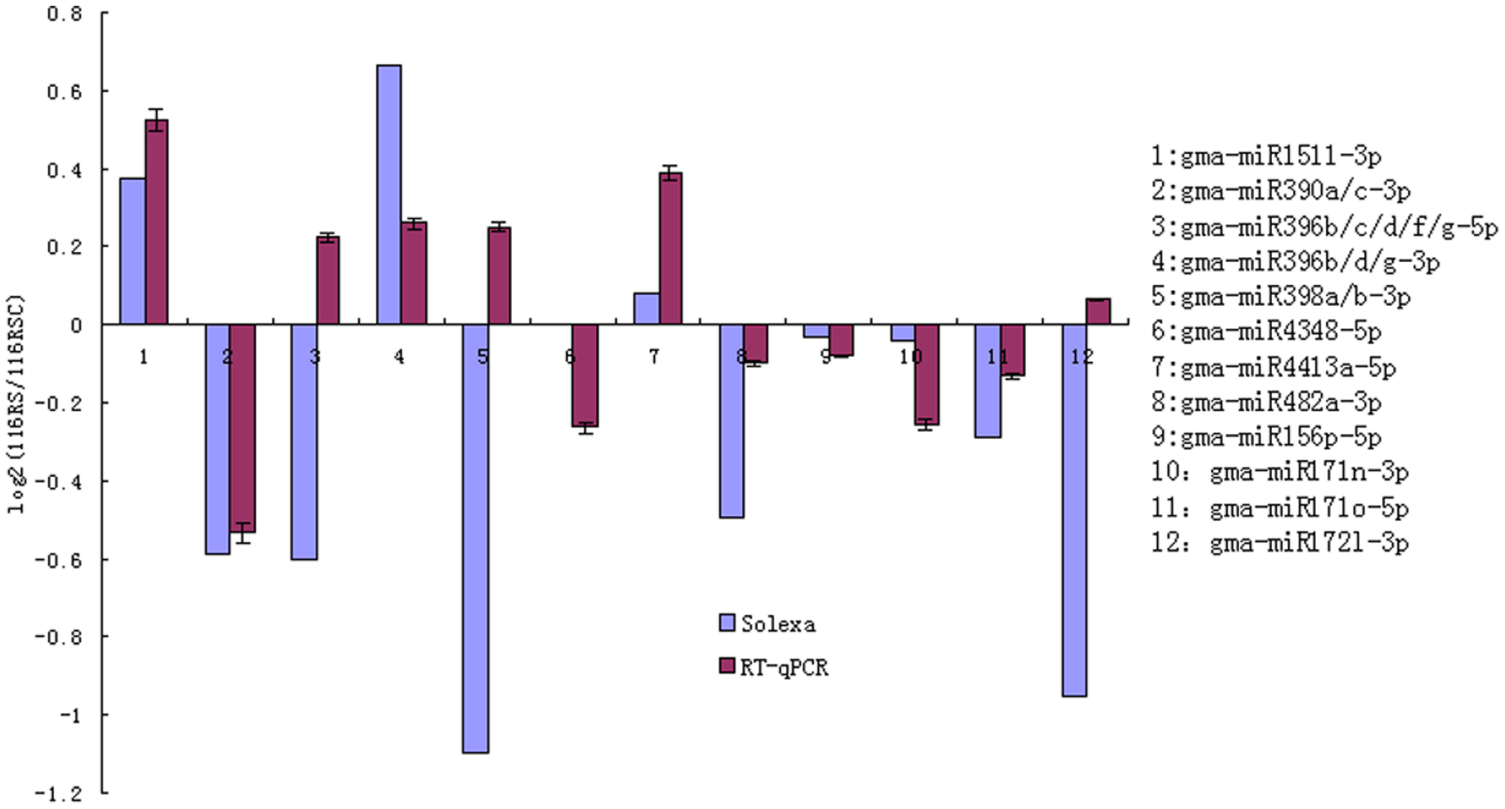
Comparison between qRT-PCR and the deep sequencing in No.116 root exposed to short-term low nitrogen stress. The y-axis indicate the relative expression levels of twelve selected miRNA in qRT-PCR and in Solexa sequencing analysis. The x-axis indicates twelve selected miRNAs, which are respectively as follows: 1. gma-miR1511-3p; 2. gma-miR390a/c-3p; 3. gma-miR396b/c/d/f/g-5p; 4. gma-miR396b/d/g-3p; 5. gma-miR398a/b-3p; 6. gma-miR4348-5p; 7. gma-miR4413a-5p; 8. gma-miR482a-3p; 9. gma-miR156p-5p; 10. gma-miR171n-3p; 11. gma-miR171o-5p; 12. gma-miR172l-3p. gma-miR1520d-3p and gma-miR156b-5p was chosen as endogenous reference genes.

After determining reference genes, 12 miRNAs were randomly selected for RT-qPCR assays to validate the reliability of Solexa/Illumina sequencing technology. Student’s t-test was used for statistical analysis of RT-qPCR data, and 12 miRNAs randomly selected did not display differential expression in response to short-term low N stress in No. 116 root.Most of RT-qPCR results were found to be consistent with the deep sequencing data (Figure 6). However, there were some differences between the deep sequencing results and RT-qPCR data. For example, deep sequencing results showed that the gma-miR172l-3p was down-regulated exposed to short-term low N stress in No. 116 root and log_2_ ratio was −0.95, while the RT-qPCR indicated that it showed negligible expression differences and log2 ratio was 0.06.

### The Targets of Low N-responsive miRNAs

To understand the functions of low N-responsive miRNAs, the identification of their targets is an important step. Since plant miRNAs are highly complementary to their targets [34], bioinformatics methods based on the homology between miRNAs and target genes are used to predict target genes and have been applied in a number of studies [69]–[72]. In this study, potential target genes of all low N–responsive miRNAs were predicted, along with the description of the function of these genes (Tables S5). We predicted a total of 223 targets genes for 53 out of all 68 low N–responsive known miRNAs as well as 14 genes for one new miRNAs in roots from the two varieties, with the remaining known miRNAs having no target genes. However, in shoots, we identified a total of 399 target genes for 93 out of all 124 low N–responsive known miRNAs, with the remaining 31 known miRNAs and the new miRNA having no target genes. While a few miRNAs were predicted to target only one gene, the great majority of low N–responsive miRNAs had multiple potential target genes. For instance, gma-miR2109-5p had the most 48 target genes followed by gma-miR156b/f-5p and gma-miR397a/b-5p with 32 and 31 target genes respectively. In general, multiple members of some miRNAs families can target the same gene, however, species from different miRNAs families might also target the same gene and thus have similar functions. For example, Glyma13g04030.1 was predicted to be the target of both gma-miR159a/e-3p-1 and gma-miR319g/l-3p, and Glyma16g05900.1 was regulated by both gma-miR156b/f-5p and gma-miR169o/r-5p. However, it was not clear how these miRNAs regulate the same genes.

To elucidate the functions of low N-responsive miRNAs, target genes with functional annotations were analyzed. We found that the predicted targets were involved in a broad range of plant physiological and biochemical processes, including regulation of protein degradation (26S proteasome regulatory complex, Apoptotic ATPase, Vesicle coat complex COPI, Mitochondrial import inner membrane translocase etc), cellular Transport (multicopper oxidase, Amino acid transporters), hormone signaling pathways (AUX/IAA family, Auxin response factor, gibberellin 2-oxidase), Carbohydrate metabolism(Long-chain acyl-CoA synthetases, acetyl-CoA carboxylase carboxyl transferase subunit, pyruvate dehydrogenase E1 component), nucleic acid metabolism(Asparaginyl-tRNA synthetase, CCAAT-binding factor, tRNA delta(2)-isopentenylpyrophosphate transferase, RNA recognition motif, Chromatin assembly factor-I). Interestingly, some target genes are also important transcription factors (Myb superfamily, TCP family transcription factor, GRAS family transcription factor, AP2 domain-containing transcription factor).

## Discussion

Extensive studies on the molecular basis underlying adaptive responses to low N stress have been conducted and many genes have been identified to be responsible for low N adaptability. For example, 10,422 genes were found to be involved in early stage responses to low N stress in rice seedling by Lian et al. [73]. However, most studies were about gene expression regulation at the transcriptional levels. With regard to post-transcriptional regulation, miRNAs associated with low N stress response in some plants have been fragmentarily reported, but little information on the post-transcriptional regulation of N limitation in soybean was available. Particularly, no miRNA in soybean has been identified to be involved in response to low N stress. In the present study, we constructed sixteen libraries for the genome-wide identification of miRNAs in soybean shoots and roots in two different genotypes exposed to long-term and short-term low N-stress using the high-throughput sequencing technology (Illumina-Solexa). We obtained 348,651,354 total reads and 52,351,387 unique reads. These data were much more than those reported in previous studies and allowed us to analyze low abundance miRNAs and identify more new miRNAs [43]–[48]. Although 508 soybean miRNAs have been well registered in miRBase database [74], 90 novel miRNAs were detected. Furthermore, sixteen constructed libraries could be compared to improve the reliability of identified miRNAs. For example, the most abundant variants of some miRNAs on their corresponding pre-miRNA were found to be different from their registered miRNA sequences, however, they were the same in all or most libraries and could be utilized to refine miRBase annotations of soybean miRNAs. In addition, the use of latest *Glycine max* genomic database and miRBase in the present study contributed to the identification of more miRNAs.

Some documents reported that miRNAs were involved in plants responses to N availability [28]–[30]. In our research, a total of 150 known miRNAs variants as well as 2 novel miRNAs were identified to be responsive to low N stress. We further analyzed the differences between the miRNAs determined in our study and the ones previously reported. For example, Gifford ML et al. found that high N repressed miR167a and resulted in the ARF8 transcript to accumulate in the pericycle to regulate root architecture [28]. In our research, all species of gma-miR167 family did not show significantly differential expression in the two varieties under long-term and short-term N limitation. Another study indicated that miR156 was up-regulated by low N in Arabidopsis, whereas miR169, miR395 and miR398 were down-regulated [29]. We found that multiple members of the gma-miR169 family were repressed in both roots and shoots of these two soybean varieties under low N stress, and some members of gma-miR398 family were down-regulated only in soybean shoots, while gma-miR395 family were not responsive to low N stress. Moreover, we also found that different species of the gma-miR156 family showed different response patterns. For example, gma-miR156b-5p and gma-miR156f-5p were repressed in roots of No.84-70 variety under long-term N limitation, and gma-miR1560-3p was up-regulated in its shoots under long-term N limitation. Recently, Xu et al. studied detailed response of miRNAs to low N availability in maize shoots and roots at the whole genome level and found that under long-term low N condition, miR167, miR169, miR395, miR399, miR408, and miR528 were down-regulated in maize roots, and in maize leaves miR164, miR172, and miR827 were up-regulated while miR169, miR397, miR398, miR399, miR408, and miR528 were down-regulated. Under short-term low N condition, miR160, miR168, miR169, miR319, miR395, and miR399 were up-regulated in roots, while in maize leaves miR172 were up-regulated and miR397, miR398, and miR827 were down-regulated. Interestingly, different species in the miR169 family showed different expression patterns, such as miR169e/f/g/h were down-regulated, while miR169i/j/k/p were up-regulated [30]. We found that in soybean roots, gma-miR408 family were up-regulated in response to long-term low N, and some species of gma-miR160 and gma-miR319 family were down-regulated in response to short-term low N. However, members of gma-miR167 and gma-miR168 were not responsive, which were contrary to the results obtained from research of Xu et al. [30]. In soybean shoots, some species of gma-miR397, gma-miR398 and gma-miR408-5p family were found to be down-regulated in response to long -term low N, and gma-miR398c-5p was found to be down-regulated in response to short -term low N.These results were consistent with the results obtained from research of Xu et al. [30], but gma-miR164, gma-miR172, gma-miR528 and gma-miR827 did not show significantly differential expression under long-term and short-term N limitation, which were different from the results obtained from research of Xu et al. [30]. Overall, Several possible explanations may account for the differences between our findings and those reported by Xu et al. [30]. The miRNAs were identified by microarray system in the study of Xu et al., which is not as sensitive and sufficient as high-throughput sequencing technology used in our study. Furthermore, the statistical methods for differentially expressed miRNA and material in our research were different from that of Xu et al [30].

One purpose of our study was to identify those miRNAs that showed different expression patterns in the two soybean varieties. Many differentially expressed miRNAs were discovered in our study and could be divided into three types. The first type was the miRNAs that showed differential expression under low N stress only in No.116 variety or No.84-70 variety. For example, gma-miR2606a/b-3p was repressed only in No.116 variety roots under short-term low N, while gma-miR1512a-5p was repressed only in No.84-70 variety roots under short-term low N. The second type of miRNAs exhibited differential expression in both soybean varieties under same low N stress, but the directions of differential expression were different. For example, gma-miR396b/c/d/f/g-5p was down-regulated in No.116 variety shoots and up-regulated in No.84-70 variety shoots under short-term low N stress. The third type of miRNAs included the miRNAs that exhibited specific differential expression under different-term low N stress in the same organ of the two varieties. For example, gma-miR319g/l-3p were down-regulated in No.84-70 variety roots under short-term low N stress while they were up-regulated in No.116 variety roots under long-term low N stress. This implied that those genotype-specific regulated miRNAs might be responsible for differences in the response of the two varieties to low N stress.

By comparing the target genes of differentially expressed miRNAs with previously discovered genes involved in low N stress response in other plants, we found that these responsive genes were indeed involved in various metabolic and regulatory pathways. For example, Peng found 21 genes involved in protein degradation through autophagy and ubiquitin-proteosome pathways were up-regulated by N limitation [75]. Some of our predicted target genes were determined to play roles in protein degradation, including Glyma07g31580 (target of gma-miR156b/6f-5p), as well as Glyma05g20930 and Glyma06g18790 (target of gma-miR396b∼g-5p), which were predicted to encode separately E3 ubiquitin ligase and Cathepsin L1. Metabolism of N is closely related to carbon metabolism, because nitrate assimilation and biosynthesis of nitrogenous macromolecules require abundant energy, reducing equivalents and organic carbon intermediates provided by carbon metabolism. Some studies found that genes involved in carbon metabolism were responsive to low N stress [75]–[76]. In the present study, we found that some targets of gma-miR159d-3p, gma-miR396b∼g-5p, such as Glyma05g23280, Glyma07g05550, Glyma16g02090, Glyma17g16750, Glyma19g44930, Glyma15g08010 and Glyma19g01200 were related to carbon metabolism. Gene expression alterations caused by low N stress correspondingly activate signal transduction and gene transcription regulatory networks to coordinate all of these changes [73], [75]–[76]. We observed that some target genes were transcription factors and participated in signal transduction, such as MYB, AP2, ARF, SPB, and zinc finger. Futhermore, MYB, ARF and AP2 families have been known to be involved in plant stress responses [77]–[79]. In addition, we noticed that gma-miR5372-5p showed distinct expression patterns between the shoots of the two genotypes under the short-term low N condition, and its target gene, Glyma09g02600, encodes peroxidase protein which could eliminate excess concentrations of reactive oxygen species produced under stress conditions. This phenomenon also was observed by Kulcheski et al who found that MIR-Seq11 had different expression behavior between the two contrasting soybean genotypes under the drought stress, and MIR-Seq11 was also predicted to target peroxidase protein [80].

In summary, our study has identified 362 known miRNAs belonging to 158 families and 90 new miRNAs belonging to 55 families from two soybean genotypes, and analyzed their expression patterns during short-term and long-term N limitation. 150 known miRNAs variants as well as 2 novel miRNAs with with significantly differential expression were discovered and the putative targets of these miRNAs were predicted. This work can contribute to a better understanding of the genetic basis of the phenotypic differences between the two soybean genotypes under N-limiting conditions and significantly contribute to future research. Our further research plans include the characterization of these differentially expressed miRNAs and their targets and understanding the complex gene regulatory network of these miRNAs.

## Supporting Information

Figure S1 Examples where unique sequences were aligned with known pre-miRNAs of soybean.
(DOC)

Figure S2 **Stability evaluation of candidate reference genes expression with geNorm software.**
(XLS)

Table S1 **Stem-loop RT-PCR Primers.** All primers used in stem-loop RT-PCR.
(XLS)

Table S2 Known miRNAs or miRNA* variants analyzed in ***Glycine max*** **.** Most abundant variants on the whole stem of known miRNA precursors (5′ arm or 3′ arm) in all libraries, comparison between unified known miRNAs variants and annotated known miRNAs and abundance of known miRNAs or miRNA* variants.
(XLS)

Table S3 **Novel miRNAs identified in** ***Glycine max*** **.** Novel miRNAs sequence in all libraries, characteristics of novel miRNAs identified and abundance of novel miRNAs.
(XLS)

Table S4 **Differential expression profiles of** ***Glycine max*** **miRNAs.** Differentially expressed unified known miRNAs variants and new miRNAs in different compared libraries response to short-term or long-term low N stress.
(XLS)

Table S5 **Predicted target genes of** ***Glycine max*** **miRNAs.** Target genes of differential expressed unified known miRNAs variants and new miRNAs in different libraries.
(XLS)
