# Supplementary material for: Elucidation of miRNAs-Mediated Responses to Low Nitrogen Stress by Deep Sequencing of Two Soybean Genotypes
Source: PLoS One. 2013 Jul 8;8(7):e67423. doi: 10.1371/journal.pone.0067423 (PMC3704600; doi:10.1371/journal.pone.0067423)
Supplement: Figure S1 — Examples where unique sequences were aligned with known pre-miRNAs of soybean. (DOC) [file pone.0067423.s001.doc]

**Figure S1.** **Examples where unique sequences were aligned with known pre-miRNAs of soybean**

**A.Examples where the most abundant variants were different among libraries and sequenced reads of them varied greatly in the same library** (only sequences with more than 10 reads are shown except the most abundant variants compared, the sequence that was the most abundant variants in the example library and another sequence was the most abundant variants in the other libraries are shown in red and blue)

**1. gma-miR1510a-5p**

>gma-MIR1510a (from 116RL)

TTATGGAACTGGAGGGATAGGTAAAACAATGACTGCTGTATAAGTAATTGTTATAGTTAGTTGTTGTTTTACCTATTCCACCCATTCCATGTA gma-MIR1510a 93

.(((((((..((.((((((((((((((((..((((((((((((....))).))))).))))..)))))))))))))))).))..))))))).. structure -44.80

************AGGGATAGGTAAAACAATGACTGC********************************************************* gma-miR1510a-5p 24

************************************************************TTGTTGTTTTACCTATTCCACCC********** gma-miR1510a-3p 23

...TGGAACTGGAGGGATAGGTAA..................................................................... t0003379 21 593

...TGGAACTGGAGGGATAGGTAAA.................................................................... t0003435 22 583

.........TGGAGGGATAGGTAAAACA................................................................. t0036659 19 35

............AGGGATAGGTAAAACAATGAC............................................................ t0006709 21 279

............AGGGATAGGTAAAACAATG.............................................................. t0070650 19 17

............AGGGATAGGTAAAACAATGA............................................................. t0103073 20 11

............................................................TTGTTGTTTTACCTATTCCAC............ t0014700 21 108

.............................................................TGTTGTTTTACCTATTCCACC........... t0001878 21 1032

.............................................................TGTTGTTTTACCTATTCCACCC.......... t0005028 22 387

.............................................................TGTTGTTTTACCTATTCCAC............ t0007814 20 234

.............................................................TGTTGTTTTACCTATTCCA............. t0074764 19 16

...............................................................TTGTTTTACCTATTCCACCCAT........ t0027175 22 51

>gma-MIR1510a (from 116RS)

TTATGGAACTGGAGGGATAGGTAAAACAATGACTGCTGTATAAGTAATTGTTATAGTTAGTTGTTGTTTTACCTATTCCACCCATTCCATGTA gma-MIR1510a 93

.(((((((..((.((((((((((((((((..((((((((((((....))).))))).))))..)))))))))))))))).))..))))))).. structure -44.80

************AGGGATAGGTAAAACAATGACTGC********************************************************* gma-miR1510a-5p 24

************************************************************TTGTTGTTTTACCTATTCCACCC********** gma-miR1510a-3p 23

...TGGAACTGGAGGGATAGGTAAA.................................................................... t0002361 22 703

...TGGAACTGGAGGGATAGGTAA..................................................................... t0002579 21 645

.........TGGAGGGATAGGTAAAACA................................................................. t0018917 19 61

.........TGGAGGGATAGGTAAAACAA................................................................ t0040063 20 24

............AGGGATAGGTAAAACAATGAC............................................................ t0004138 21 386

............AGGGATAGGTAAAACAATG.............................................................. t0022720 19 49

............AGGGATAGGTAAAACAATGA............................................................. t0035858 20 28

............AGGGATAGGTAAAACAATGACT........................................................... t0071258 22 13

............................................................TTGTTGTTTTACCTATTCCAC............ t0021881 21 51

.............................................................TGTTGTTTTACCTATTCCACC........... t0002927 21 563

.............................................................TGTTGTTTTACCTATTCCACCC.......... t0006702 22 222

.............................................................TGTTGTTTTACCTATTCCAC............ t0013296 20 95

.............................................................TGTTGTTTTACCTATTCC.............. t0078519 18 11

...............................................................TTGTTTTACCTATTCCACCCAT........ t0029099 22 36

**2. gma-miR398b-5p**

>gma-MIR398b (from 116RL)

AGTCCAAATGGTTTATCTCAGAGGAGTGGATCTGAGAACACAAGGCTGGTTTGCACTGCTATATTATGATCGATTGGTATAAGGTGAATTTACTTTGTGTTCTCAGGTCACCCCTTTGAGCCAACCTGTTGACAT gma-MIR398b 135

.(((.....((((...((((((((.(((.((((((((((((((((..((((..(.((..(((((((........))))))))))..))))..))))))))))))))))))).))))))))..))))....))).. structure -56.90

***********************************************************************************************TGTGTTCTCAGGTCACCCCTT******************* gma-miR398b 21

.................TCAGAGGAGTGGATCTGAGAAC................................................................................................ t0030596 22 44

.................TCAGAGGAGTGGATCTGAGAA................................................................................................. t0070815 21 17

..................CAGAGGAGTGGATCTGAGAACA............................................................................................... t0011182 22 151

......................GGAGTGGATCTGAGAACACAAG........................................................................................... t0000346 22 4149

......................GGAGTGGATCTGAGAACACAAGG.......................................................................................... t0015602 23 101

......................GGAGTGGATCTGAGAACACAA............................................................................................ t0093339 21 12

.......................GAGTGGATCTGAGAACACAAGG.......................................................................................... t0001900 22 1020

.......................GAGTGGATCTGAGAACACAAG........................................................................................... t0012272 21 135

.......................GAGTGGATCTGAGAACACAAGGC......................................................................................... t0097680 23 12

.......................GAGTGGATCTGAGAACACAAGGCTGGTTT................................................................................... t4693413 29 1

........................AGTGGATCTGAGAACACAAGGC......................................................................................... t0000619 22 2677

........................AGTGGATCTGAGAACACAAGGCT........................................................................................ t0007374 23 251

........................AGTGGATCTGAGAACACAAGG.......................................................................................... t0035166 21 37

.........................GTGGATCTGAGAACACAAGGC......................................................................................... t0029070 21 47

..........................TGGATCTGAGAACACAAGGC......................................................................................... t0100299 20 11

.............................................................................................TTTGTGTTCTCAGGTCACCCC..................... t0061310 21 19

..............................................................................................TTGTGTTCTCAGGTCACCCCT.................... t0014096 21 114

...................................................................................................TTCTCAGGTCACCCCTTTGAG............... t0006603 21 285

>gma-MIR398b (from 116SL)

AGTCCAAATGGTTTATCTCAGAGGAGTGGATCTGAGAACACAAGGCTGGTTTGCACTGCTATATTATGATCGATTGGTATAAGGTGAATTTACTTTGTGTTCTCAGGTCACCCCTTTGAGCCAACCTGTTGACAT gma-MIR398b 135

.(((.....((((...((((((((.(((.((((((((((((((((..((((..(.((..(((((((........))))))))))..))))..))))))))))))))))))).))))))))..))))....))).. structure -56.90

***********************************************************************************************TGTGTTCTCAGGTCACCCCTT******************* gma-miR398b 21

..................CAGAGGAGTGGATCTGAGAACA............................................................................................... t0008252 22 37

......................GGAGTGGATCTGAGAACACAAG........................................................................................... t0001765 22 167

.......................GAGTGGATCTGAGAACACAAGG.......................................................................................... t0002412 22 126

........................AGTGGATCTGAGAACACAAGGC......................................................................................... t0000617 22 472

........................AGTGGATCTGAGAACACAAGGCT........................................................................................ t0018273 23 16

.............................................................................................TTTGTGTTCTCAGGTCACCCC..................... t0028778 21 10

..............................................................................................TTGTGTTCTCAGGTCACCCCT.................... t0021260 21 14

...................................................................................................TTCTCAGGTCACCCCTTTGAG............... t0002981 21 104

**3.gma-miR1511-3p**

>gma-MIR1511(from 116RL)

TCAGCCGTGGTATCAGGTCCTGCTTCATCAAGTGGTCTTGTGTTCAAATCCAGCCTCAAGCACATGGTTAACCAGGCTCTGATACCATGGTGAATATAA gma-MIR1511 99

(((.((((((((((((..((((.((.((((.(((..((((.(((.......)))..))))))).)))).)).))))..)))))))))))))))...... structure -38.60

*********************************************************************AACCAGGCTCTGATACCATG********** gma-miR1511 20

......GTGGTATCAGGTCCTGCTTCA........................................................................ t0008586 21 209

......GTGGTATCAGGTCCTGCTT.......................................................................... t0106152 19 11

....................................................................TAACCAGGCTCTGATACCA............ t0094752 19 12

.....................................................................AACCAGGCTCTGATACCATG.......... t0038969 20 33

.....................................................................AACCAGGCTCTGATACCA............ t0054702 18 22

.....................................................................AACCAGGCTCTGATACCAT........... t0116887 19 10

.....................................................................AACCAGGCTCTGATACCATGG......... t0208059 21 5

>gma-MIR1511 (from 116RS)

TCAGCCGTGGTATCAGGTCCTGCTTCATCAAGTGGTCTTGTGTTCAAATCCAGCCTCAAGCACATGGTTAACCAGGCTCTGATACCATGGTGAATATAA gma-MIR1511 99

(((.((((((((((((..((((.((.((((.(((..((((.(((.......)))..))))))).)))).)).))))..)))))))))))))))...... structure -38.60

*********************************************************************AACCAGGCTCTGATACCATG********** gma-miR1511 20

......GTGGTATCAGGTCCTGCTTCA........................................................................ t0005617 21 274

......GTGGTATCAGGTCCTGCTTC......................................................................... t0072121 20 12

...................................................................TTAACCAGGCTCTGATACCAT........... t0033822 21 30

.....................................................................AACCAGGCTCTGATACCATGG......... t0010517 21 127

.....................................................................AACCAGGCTCTGATACCATG.......... t0038774 20 25

**4.gma-miR166h-3p**

>gma-MIR166h (from 116RL)

TGGGGATGATGGGAATGTTGTTTGGCTCGAGGTAACTGCATGGTCTTAATTTTGTTCATCTTTTGAAGCTTTAATTTATTTATGGGTTTCAATCTTTTTTGATCCCTTGAAACAGAAAAAGCTTTAAAGGTTGGATTTTGAGGCTATCCCTTTATGTGATCTCGGACCAGGCTTCATTCCCGTCAACCTT gma-MIR166h 190

.((((.(((((((((((..((((((..((((((.((.(.((((((((((....(((((.(((((((((((((......((((.(((..((((......)))).))).))))......))))))))))))).))))).)))))))))).)......)).))))))..))))))..))))))))))).)))) structure -76.30

***********GGAATGTTGTTTGGCTCGAGG************************************************************************************************************************************************************** gma-miR166h-5p 21

***************************************************************************************************************************************************************TCTCGGACCAGGCTTCATTCC********** gma-miR166h-3p 21

.....ATGATGGGAATGTTGTTTGGC.................................................................................................................................................................... t0076098 21 15

...........GGAATGTTGTTTGGCTCGAGG.............................................................................................................................................................. t0019502 21 76

...........GGAATGTTGTTTGGCTCGAGGT............................................................................................................................................................. t0368832 22 3

...............................................................................................................................................................TCTCGGACCAGGCTTCATTCC.......... t0000010 21 86641

...............................................................................................................................................................TCTCGGACCAGGCTTCATTC........... t0000139 20 9008

...............................................................................................................................................................TCTCGGACCAGGCTTCATT............ t0002777 19 720

...............................................................................................................................................................TCTCGGACCAGGCTTCAT............. t0014434 18 110

...............................................................................................................................................................TCTCGGACCAGGCTTCATTCCC......... t0030599 22 44

................................................................................................................................................................CTCGGACCAGGCTTCATTCCC......... t0010693 21 160

................................................................................................................................................................CTCGGACCAGGCTTCATTCC.......... t0012859 20 128

................................................................................................................................................................CTCGGACCAGGCTTCATTCCCG........ t0086696 22 13

................................................................................................................................................................CTCGGACCAGGCTTCATTC........... t0111248 19 10

.................................................................................................................................................................TCGGACCAGGCTTCATTCCCG........ t0000016 21 55964

.................................................................................................................................................................TCGGACCAGGCTTCATTCC.......... t0000598 19 2744

.................................................................................................................................................................TCGGACCAGGCTTCATTCCC......... t0001055 20 1746

.................................................................................................................................................................TCGGACCAGGCTTCATTC........... t0003885 18 511

.................................................................................................................................................................TCGGACCAGGCTTCATTCCCGT....... t0007205 22 257

..................................................................................................................................................................CGGACCAGGCTTCATTCCCG........ t0025633 20 54

...................................................................................................................................................................GGACCAGGCTTCATTCCCG........ t0018116 19 84

>gma-MIR166h (from 116SS)

TGGGGATGATGGGAATGTTGTTTGGCTCGAGGTAACTGCATGGTCTTAATTTTGTTCATCTTTTGAAGCTTTAATTTATTTATGGGTTTCAATCTTTTTTGATCCCTTGAAACAGAAAAAGCTTTAAAGGTTGGATTTTGAGGCTATCCCTTTATGTGATCTCGGACCAGGCTTCATTCCCGTCAACCTT gma-MIR166h 190

.((((.(((((((((((..((((((..((((((.((.(.((((((((((....(((((.(((((((((((((......((((.(((..((((......)))).))).))))......))))))))))))).))))).)))))))))).)......)).))))))..))))))..))))))))))).)))) structure -76.30

***********GGAATGTTGTTTGGCTCGAGG************************************************************************************************************************************************************** gma-miR166h-5p 21

***************************************************************************************************************************************************************TCTCGGACCAGGCTTCATTCC********** gma-miR166h-3p 21

...........GGAATGTTGTTTGGCTCGAGG.............................................................................................................................................................. t0017828 21 47

..............................................................................................................................................................ATCTCGGACCAGGCTTCATTC........... t0065964 21 13

...............................................................................................................................................................TCTCGGACCAGGCTTCATTCC.......... t0000049 21 12659

...............................................................................................................................................................TCTCGGACCAGGCTTCATTC........... t0000627 20 909

...............................................................................................................................................................TCTCGGACCAGGCTTCATT............ t0015156 19 56

...............................................................................................................................................................TCTCGGACCAGGCTTCAT............. t0079996 18 10

................................................................................................................................................................CTCGGACCAGGCTTCATTCCC......... t0001401 21 480

................................................................................................................................................................CTCGGACCAGGCTTCATTCC.......... t0046998 20 18

.................................................................................................................................................................TCGGACCAGGCTTCATTCCCG........ t0000037 21 20268

.................................................................................................................................................................TCGGACCAGGCTTCATTCC.......... t0000267 19 1938

.................................................................................................................................................................TCGGACCAGGCTTCATTCCC......... t0000292 20 1816

.................................................................................................................................................................TCGGACCAGGCTTCATTC........... t0001425 18 475

.................................................................................................................................................................TCGGACCAGGCTTCATTCCCGT....... t0006981 22 117

..................................................................................................................................................................CGGACCAGGCTTCATTCCCG........ t0030770 20 27

...................................................................................................................................................................GGACCAGGCTTCATTCCCG........ t0035268 19 24

**5.gma-miR172i-3p**

>gma-MIR172i (from 116RL)

GCAGGTGCAGCAGCATCAAGATTCACACCGCCTAATTTGCTAGGACTTCAGGACTGCACACGCTTAATTATACATACATATATACATATATGTTAGCTCTTTGTGGAGTGCGGAATAAAGTTCTATTTTAGATGTGGGAATCTTGATGATGCTGCATCAGC gma-MIR172i 161

((.(((((((((.(((((((((((.(((...((((.....((((((((.(...((((((.(((............(((((((....))))))).........)))..))))))..).))))))))..))))..))).))))))))))).))))))))).)) structure -60.10

******GCAGCAGCATCAAGATTCACA************************************************************************************************************************************** gma-miR172i 21

......GCAGCAGCATCAAGATTCACA...................................................................................................................................... t0018259 21 83

......GCAGCAGCATCAAGATTCAC....................................................................................................................................... t0077786 20 15

.......CAGCAGCATCAAGATTCACAC..................................................................................................................................... t0042177 21 30

.......................................................................................................................................GGGAATCTTGATGATGCTGCA..... t0075212 21 15

.......................................................................................................................................GGGAATCTTGATGATGCTGCAT.... t0083447 22 14

........................................................................................................................................GGAATCTTGATGATGCTGCAT.... t0027761 21 49

.........................................................................................................................................GAATCTTGATGATGCTGCAT.... t0115726 20 10

>gma-MIR172i (from 116SS)

GCAGGTGCAGCAGCATCAAGATTCACACCGCCTAATTTGCTAGGACTTCAGGACTGCACACGCTTAATTATACATACATATATACATATATGTTAGCTCTTTGTGGAGTGCGGAATAAAGTTCTATTTTAGATGTGGGAATCTTGATGATGCTGCATCAGC gma-MIR172i 161

((.(((((((((.(((((((((((.(((...((((.....((((((((.(...((((((.(((............(((((((....))))))).........)))..))))))..).))))))))..))))..))).))))))))))).))))))))).)) structure -60.10

******GCAGCAGCATCAAGATTCACA************************************************************************************************************************************** gma-miR172i 21

.....TGCAGCAGCATCAAGATTCACA...................................................................................................................................... t0995834 22 1

......GCAGCAGCATCAAGATTCACA...................................................................................................................................... t0003318 21 229

......GCAGCAGCATCAAGATTCAC....................................................................................................................................... t0014707 20 57

........................................................................................................................................GGAATCTTGATGATGCTGCAT.... t0175067 21 5

.........................................................................................................................................GAATCTTGATGATGCTGCAT.... t0015598 20 54

**6.gma-MIR4361-3p**

>gma-MIR4361 (from 116RL)

CAAGTTGATCCGGAAGTCTCTTACGGATCAAGTTGATCCGGAAGAGACTTACGGATCAACTTA gma-MIR4361 63

.(((((((((((.(((((((((.((((((.....)))))).))))))))).))))))))))). structure -41.00

*************************************CCGGAAGAGACTTACGGATCAACT** gma-miR4361 24

.....................................CCGGAAGAGACTTACGGATCAA.... t0112236 22 10

.....................................CCGGAAGAGACTTACGGATCAACT.. t1497812 24 1

.........................................AAGAGACTTACGGATCAACTT. t0087591 21 13

>gma-MIR4361(from 84RL)

CAAGTTGATCCGGAAGTCTCTTACGGATCAAGTTGATCCGGAAGAGACTTACGGATCAACTTA gma-MIR4361 63

.(((((((((((.(((((((((.((((((.....)))))).))))))))).))))))))))). structure -41.00

*************************************CCGGAAGAGACTTACGGATCAACT** gma-miR4361 24

.....................................CCGGAAGAGACTTACGGATCAACT.. t0059869 24 20

.........................................AAGAGACTTACGGATCAACTT. t0312688 21 4

**7.gma-MIR4368b-3p**

>gma-MIR4368b (from 116RL)

CAAGGACGGTACTTACGTAAGCAACGTCTTTGAAAGTTCACAAACAAAGACGGTGCAAGCAACGTCCACAAACAAAGACGGTGCAAGCAACGTCGTATTTGTTTGTGAACTTTCAAAGACGCTGCTTATGTAAGCACCGTCTTTT gma-MIR4368b 145

.(((((((((.((((((((((((.((((((((((((((((((((((((((((.(((..(((.((((..........)))).)))..))).))))...)))))))))))))))))))))))).)))))))))))).))))))))). structure -84.30

*AAGGACGGTACTTACGTAAGCAAC************************************************************************************************************************ gma-miR4368b 24

.................................................................................................TTTGTTTGTGAACTTTCAAAGA.......................... t0040187 22 32

......................................................................................................TTGTGAACTTTCAAAGACGCTGCT................... t0288409 24 4

>gma-MIR4368b (from 84RL)

CAAGGACGGTACTTACGTAAGCAACGTCTTTGAAAGTTCACAAACAAAGACGGTGCAAGCAACGTCCACAAACAAAGACGGTGCAAGCAACGTCGTATTTGTTTGTGAACTTTCAAAGACGCTGCTTATGTAAGCACCGTCTTTT gma-MIR4368b 145

.(((((((((.((((((((((((.((((((((((((((((((((((((((((.(((..(((.((((..........)))).)))..))).))))...)))))))))))))))))))))))).)))))))))))).))))))))). structure -84.30

*AAGGACGGTACTTACGTAAGCAAC************************************************************************************************************************ gma-miR4368b 24

.................................................................................................TTTGTTTGTGAACTTTCAAAGA.......................... t0935512 22 2

......................................................................................................TTGTGAACTTTCAAAGACGCTGCT................... t0093028 24 13

**B. Examples where miRNA variants were more consistent than miRNA* variants among among libraries** (only sequences with more than 10 reads are shown except the most abundant variants compared, the sequence that was the most abundant variants in the example library and another sequence was the most abundant variants in the other libraries are shown in red and blue)

**1. gma-MIR167g**

>gma-MIR167g (from 116RS)

CAGCAGTTGAAGCTGCCAGCATGATCTGAGTTTACCTTCTATTGGTAAGAACAGATCATGTGGCTGCTTCACCTGTTG gma-MIR167g 78

((((((.((((((.((((.(((((((((..((((((.......))))))..))))))))))))).)))))).)))))) structure -44.90

*******TGAAGCTGCCAGCATGATCTGA************************************************* gma-miR167g 22

.......TGAAGCTGCCAGCATGATCTG.................................................. t0000280 21 4559

.......TGAAGCTGCCAGCATGATCTGA................................................. t0007203 22 202

.......TGAAGCTGCCAGCATGATCT................................................... t0007723 20 186

.......TGAAGCTGCCAGCATGATC.................................................... t0047061 19 20

..........AGCTGCCAGCATGATCTGAGTT.............................................. t0032142 22 32

.................................................AACAGATCATGTGGCTGCTTCACC..... t0074393 24 12

.................................................AACAGATCATGTGGCTGCTTC........ t0525995 21 2

**2. gma-MIR167g**

>gma-MIR167g (from 116RSC)

CAGCAGTTGAAGCTGCCAGCATGATCTGAGTTTACCTTCTATTGGTAAGAACAGATCATGTGGCTGCTTCACCTGTTG gma-MIR167g 78

((((((.((((((.((((.(((((((((..((((((.......))))))..))))))))))))).)))))).)))))) structure -44.90

*******TGAAGCTGCCAGCATGATCTGA************************************************* gma-miR167g 22

......TTGAAGCTGCCAGCATGATCT................................................... t0080685 21 11

.......TGAAGCTGCCAGCATGATCTG.................................................. t0000184 21 6279

.......TGAAGCTGCCAGCATGATCT................................................... t0004561 20 324

.......TGAAGCTGCCAGCATGATCTGA................................................. t0004934 22 296

.......TGAAGCTGCCAGCATGATC.................................................... t0039053 19 24

.......TGAAGCTGCCAGCATGATCTGAGTT.............................................. t0068761 25 13

..........AGCTGCCAGCATGATCTGAGTT.............................................. t0021960 22 49

.................................................AACAGATCATGTGGCTGCTTC........ t0067670 21 13

.................................................AACAGATCATGTGGCTGCTTCACC..... t0096963 24 9

**3. gma-MIR169f**

>gma-MIR169f (from 116SS)

GAACTTGCACGAAGAGGCAGAGAGTGTAGTGCAGCCAAGGATGACTTGCCGGCATTAGCCAAGTGAATGAGCATCATATATATATATATATATATATATATATATATGACTCATGTTCTTGTCGGCAAGTTGGCCTTGGCTATATTGGACTCTCTTCTTTTCATGTAAGT gma-MIR169f 170

..(((((((.(((((((.(((((((.(((((.((((((((..((((((((((((..(((.......(((((..((((((((((((((((....))))))))))))))))))))))))..))))))))))))..)))))))).))))).)))))))))))))).))))))) structure -87.71

*******************************CAGCCAAGGATGACTTGCCGG********************************************************************************************************************** gma-miR169f 21

.........CGAAGAGGCAGAGAGTGTAGT............................................................................................................................................ t0036898 21 22

.........CGAAGAGGCAGAGAGTGTAGTG........................................................................................................................................... t0052347 22 16

...............................CAGCCAAGGATGACTTGCCGG...................................................................................................................... t0000578 21 971

...............................CAGCCAAGGATGACTTGCCGGCAT................................................................................................................... t0077745 24 11

................................AGCCAAGGATGACTTGCCGG...................................................................................................................... t0013263 20 64

...................................CAAGGATGACTTGCCGGCATT.................................................................................................................. t0002967 21 250

...........................................................................................................................GGCAAGTTGGCCTTGGCTATA.......................... t0010119 21 83

...........................................................................................................................GGCAAGTTGGCCTTGGCTAT........................... t0125771 20 7

**4. gma-MIR169f**

>gma-MIR169f (from 116SSC)

GAACTTGCACGAAGAGGCAGAGAGTGTAGTGCAGCCAAGGATGACTTGCCGGCATTAGCCAAGTGAATGAGCATCATATATATATATATATATATATATATATATATGACTCATGTTCTTGTCGGCAAGTTGGCCTTGGCTATATTGGACTCTCTTCTTTTCATGTAAGT gma-MIR169f 170

..(((((((.(((((((.(((((((.(((((.((((((((..((((((((((((..(((.......(((((..((((((((((((((((....))))))))))))))))))))))))..))))))))))))..)))))))).))))).)))))))))))))).))))))) structure -87.71

*******************************CAGCCAAGGATGACTTGCCGG********************************************************************************************************************** gma-miR169f 21

.........CGAAGAGGCAGAGAGTGTAGT............................................................................................................................................ t0032423 21 26

.........CGAAGAGGCAGAGAGTGTAGTG........................................................................................................................................... t0069154 22 13

...............................CAGCCAAGGATGACTTGCCGG...................................................................................................................... t0000967 21 716

................................AGCCAAGGATGACTTGCCGG...................................................................................................................... t0013136 20 65

...................................CAAGGATGACTTGCCGGCATT.................................................................................................................. t0003852 21 214

..........................................................................................................................CGGCAAGTTGGCCTTGGCTAT........................... t0032876 21 26

...........................................................................................................................GGCAAGTTGGCCTTGGCTAT........................... t0002598 20 310

...........................................................................................................................GGCAAGTTGGCCTTGGCTATA.......................... t0006415 21 133

.............................................................................................................................CAAGTTGGCCTTGGCTATATT........................ t0088210 21 10

**5. gma-MIR394b**

>gma-MIR394b (from 116RL)

TAACAGAGTTTATTGGCATTCTGTCCACCTCCACTTCCTACTCTCTCTCTGAGCCACATGTTCGTGAAGTTGGAGGTGGGCATACTGTCAACTGAGTTCTGTTG gma-MIR394b 104

(((((((..(((((((((.(.(((((((((((((((((.((.(((.....)))......))..).)))).)))))))))))).).)))))).)))..))))))) structure -42.40

*************************************************************************AGGTGGGCATACTGTCAACT*********** gma-miR394b 20

............TTGGCATTCTGTCCACCTCC........................................................................ t0123577 20 9

.........................................................................AGGTGGGCATACTGTCAACTG.......... t0175737 21 6

.........................................................................AGGTGGGCATACTGTCAACT........... t0328406 20 4

>gma-MIR394b (from 116RLC)

TAACAGAGTTTATTGGCATTCTGTCCACCTCCACTTCCTACTCTCTCTCTGAGCCACATGTTCGTGAAGTTGGAGGTGGGCATACTGTCAACTGAGTTCTGTTG gma-MIR394b 104

(((((((..(((((((((.(.(((((((((((((((((.((.(((.....)))......))..).)))).)))))))))))).).)))))).)))..))))))) structure -42.40

*************************************************************************AGGTGGGCATACTGTCAACT*********** gma-miR394b 20

............TTGGCATTCTGTCCACCTCC........................................................................ t0089567 20 11

.........................................................................AGGTGGGCATACTGTCAACT........... t0129861 20 8

.........................................................................AGGTGGGCATACTGTCAACTG.......... t0152660 21 6

**C. Examples where the alternative use of the pre-miRNAs 5’ and pre-miRNAs 3’ arms as miRNA variants** (only sequences with more than 10 reads are shown except the most abundant variants compared, the sequences that was the most abundant variants on the two arm of are pre-miRNAs were shown in red)

**1. gma-MIR169l**

>gma-MIR169l (from 84SL)

GAGTGATTTGCAGCCAAGAATGACTTGCCGGAATGCATATATATGCATTAGGTACCAACATATATAGTTGTATTGTATAATTTCGGGCAAGTTGTTTTTGGCTACATTTATCTC gma-MIR169l 114

(((.....((.(((((((((..(((((((.(((.((((....))))....(((((.(((.......)))))))).......))).)))))))..))))))))).)).....))) structure -41.30

**********CAGCCAAGAATGACTTGCCGG*********************************************************************************** gma-miR169l 21

..........CAGCCAAGAATGACTTGCCGG................................................................................... t0010114 21 39

..............CAAGAATGACTTGCCGGAATG............................................................................... t0033489 21 12

.................................................................................TTCGGGCAAGTTGTTTTTGGC............ t0021728 21 18

>gma-MIR169l (from 84RSC)

GAGTGATTTGCAGCCAAGAATGACTTGCCGGAATGCATATATATGCATTAGGTACCAACATATATAGTTGTATTGTATAATTTCGGGCAAGTTGTTTTTGGCTACATTTATCTC gma-MIR169l 114

(((.....((.(((((((((..(((((((.(((.((((....))))....(((((.(((.......)))))))).......))).)))))))..))))))))).)).....))) structure -41.30

**********CAGCCAAGAATGACTTGCCGG*********************************************************************************** gma-miR169l 21

..........CAGCCAAGAATGACTTGCCGG................................................................................... t0005367 21 270

...........AGCCAAGAATGACTTGCCGG................................................................................... t0042213 20 26

...........AGCCAAGAATGACTTGCCGGA.................................................................................. t0044498 21 25

..............CAAGAATGACTTGCCGGAATG............................................................................... t0023104 21 51

.................................................................................TTCGGGCAAGTTGTTTTTGGC............ t0034121 21 33

..................................................................................TCGGGCAAGTTGTTTTTGGCT........... t0052591 21 21

...................................................................................CGGGCAAGTTGTTTTTGGCTAC......... t0003283 22 448

...................................................................................CGGGCAAGTTGTTTTTGGCTA.......... t0009671 21 141

**2. gma-MIR171b**

>gma-MIR171b (from 84SS)

TAGACACGGCGTGATATTGGTACGGCTCATCTTAATTCAACCATTATATCAACAATTGAGACGAGCCGAATCAATATCACTCTTGTTTGCTT gma-MIR171b 92

((((((.((.(((((((((((.((((((.((((((((................)))))))).)))))).))))))))))).))))))))... structure -36.39

*****ACGGCGTGATATTGGTACGGCTC**************************************************************** gma-miR171b-5p 23

*************************************************************CGAGCCGAATCAATATCACTC********** gma-miR171b-3p 21

......CGGCGTGATATTGGTACGGCTC................................................................ t0055646 22 16

.........CGTGATATTGGTACGGCTCATC............................................................. t0003429 22 187

.........CGTGATATTGGTACGGCTCATCT............................................................ t0009282 23 80

...........TGATATTGGTACGGCTCATCT............................................................ t0056374 21 16

.............................................................CGAGCCGAATCAATATCACTC.......... t0001423 21 394

>gma-MIR171b (from 84SSC)

TAGACACGGCGTGATATTGGTACGGCTCATCTTAATTCAACCATTATATCAACAATTGAGACGAGCCGAATCAATATCACTCTTGTTTGCTT gma-MIR171b 92

((((((.((.(((((((((((.((((((.((((((((................)))))))).)))))).))))))))))).))))))))... structure -36.39

*****ACGGCGTGATATTGGTACGGCTC**************************************************************** gma-miR171b-5p 23

*************************************************************CGAGCCGAATCAATATCACTC********** gma-miR171b-3p 21

......CGGCGTGATATTGGTACGGCTC................................................................ t0026032 22 40

.........CGTGATATTGGTACGGCTCATC............................................................. t0000909 22 754

.........CGTGATATTGGTACGGCTCATCT............................................................ t0003724 23 242

.........CGTGATATTGGTACGGCTCATCTT........................................................... t0028246 24 37

.........CGTGATATTGGTACGGCTCAT.............................................................. t0038614 21 28

...........TGATATTGGTACGGCTCATCT............................................................ t0010352 21 96

.............................................................CGAGCCGAATCAATATCACTC.......... t0000953 21 728

**3. gma-MIR4414**

>gma-MIR4414 (from 84SS)

ACTGAAACTCAGCTGCTGACTCGTTGGCTCGAGAGTTCACCATTAAGATAGATTGATGTTCGGCACTCACGCCATGTAATTAAGGTGGTGTGCTTCTGATCCAACGATGCGGGAGCTGCATTCAGTCG gma-MIR4414 128

((((((...(((((.(((..(((((((.(((..(((.(((((((..((((......)))).(((......)))..........))))))).)))..))).)))))))..))).)))))..)))))).. structure -50.30

**********AGCTGCTGACTCGTTGGCTC************************************************************************************************** gma-miR4414 20

..........AGCTGCTGACTCGTTGGCTCG................................................................................................. t0003409 21 188

..........AGCTGCTGACTCGTTGGCTC.................................................................................................. t0010709 20 70

..................................................................................................ATCCAACGATGCGGGAGCTGC......... t0004178 21 159

>gma-MIR4414 (from 84SSC)

ACTGAAACTCAGCTGCTGACTCGTTGGCTCGAGAGTTCACCATTAAGATAGATTGATGTTCGGCACTCACGCCATGTAATTAAGGTGGTGTGCTTCTGATCCAACGATGCGGGAGCTGCATTCAGTCG gma-MIR4414 128

((((((...(((((.(((..(((((((.(((..(((.(((((((..((((......)))).(((......)))..........))))))).)))..))).)))))))..))).)))))..)))))).. structure -50.30

**********AGCTGCTGACTCGTTGGCTC************************************************************************************************** gma-miR4414 20

.....AACTCAGCTGCTGACTCGTTGG..................................................................................................... t0089922 22 12

......ACTCAGCTGCTGACTCGTTGG..................................................................................................... t0026608 21 39

..........AGCTGCTGACTCGTTGGCTCG................................................................................................. t0000578 21 1075

..........AGCTGCTGACTCGTTGGCTC.................................................................................................. t0000832 20 811

..................................................................................................ATCCAACGATGCGGGAGCTGC......... t0000236 21 2255

..................................................................................................ATCCAACGATGCGGGAGCTGCATT...... t0040560 24 26

..................................................................................................ATCCAACGATGCGGGAGCTGCAT....... t0057238 23 19

...................................................................................................TCCAACGATGCGGGAGCTGC......... t0003823 20 236

...................................................................................................TCCAACGATGCGGGAGCTGCA........ t0098328 21 11

**D. Examples where distinct miRNAs generated from a single precursors** (only sequences with more than 10 reads are shown except the most abundant variants compared, the sequences that was the most abundant variants on the two arm of are pre-miRNAs were shown in red)

**1. gma-MIR159a**

>gma-MIR159a (from 116SS)

AATTAAAGGGGATTATGAAGTGGAGCTCCTTGAAGTCCAATTGAGGATCTTACTGGGTGAATTGAGCTGCTTAGCTATGGATCCCACAGTTCTACCCATCAATAAGTGCTTTTGTGGTAGTCTTGTGGCTTCCATATTTGGGGAGCTTCATTTGCCTTTATAGTATTAACCTTCTTTGGATTGAAGGGAGCTCTACACCCTTCTCTTCTTTTCT gma-MIR159a 214

....((((((((.......((((((((((((..(((((((..((((....(((((((..(((.(((((.(((((.((((((..((((((..(((((((..............)).)))))..))))))..)))))).))))).))))).)))..)))....))))....))))..)))))))..)))))))))))).......))))))))... structure -89.98

******************************************************************************************************************************************************************************TTTGGATTGAAGGGAGCTCTA******************* gma-miR159a 21

......................GAGCTCCTTGAAGTCCAATTG........................................................................................................................................................................... t0071846 21 12

......................GAGCTCCTTGAAGTCCAATT............................................................................................................................................................................ t0111953 20 7

................................................................AGCTGCTTAGCTATGGATCCC................................................................................................................................. t0057424 21 14

.................................................................GCTGCTTAGCTATGGATCCC................................................................................................................................. t3088316 20 1

............................................................................................................................................................................TCTTTGGATTGAAGGGAG........................ t0394146 18 2

............................................................................................................................................................................TCTTTGGATTGAAGGGAGCTC..................... t0513413 21 2

............................................................................................................................................................................TCTTTGGATTGAAGGGAGCT...................... t0795367 20 1

.............................................................................................................................................................................CTTTGGATTGAAGGGAGCTCTA................... t1143935 22 1

.............................................................................................................................................................................CTTTGGATTGAAGGGAGC....................... t3335211 18 1

..............................................................................................................................................................................TTTGGATTGAAGGGAGCTCTA................... t0000288 21 1835

..............................................................................................................................................................................TTTGGATTGAAGGGAGCT...................... t0001882 18 374

..............................................................................................................................................................................TTTGGATTGAAGGGAGCTCT.................... t0030626 20 27

..............................................................................................................................................................................TTTGGATTGAAGGGAGCTC..................... t0035203 19 24

..............................................................................................................................................................................TTTGGATTGAAGGGAGCTCTAC.................. t2002247 22 1

...............................................................................................................................................................................TTGGATTGAAGGGAGCTCTA................... t0041748 20 20

...............................................................................................................................................................................TTGGATTGAAGGGAGCTCT.................... t1086384 19 1

................................................................................................................................................................................TGGATTGAAGGGAGCTCTA................... t0502217 19 2

**2. gma-MIR319a**

>gma-MIR319a(from 116SS)

CGTTGAAGACCCTAAGGTAAGAGAGCTTTCTTCAGTCCACTCATGGGTGACAGTAAGATTCAATTAGCTGCCGACTCATTCATCCAAATGTTGAGTGTAAGCGAATAAATATACTCAGCAGATGAGTGAATGATGCGGGAGACAAATTGAATCTTAAGTTTCCTGTACTTGGACTGAAGGGAGCTCCCTTTTCCTTTTGTCTCTTAC gma-MIR319a 207

......((((...((((.(((.(((((..((((((((((...(((((.(((..((((((((((((.(((.(((..(((((((..((..(((((((((((..........)))))))))))..))..)))))))..))).)).).)))))))))))).))).)))))...))))))))))..))))).)))..))))..))))..... structure -93.20

************************************************************************************************************************************************************************TTGGACTGAAGGGAGCTCCC******************* gma-miR319a 20

.....................AGAGCTTTCTTCAGTCCACTC..................................................................................................................................................................... t0435674 21 2

.....................AGAGCTTTCTTCAGTCCACT...................................................................................................................................................................... t2035914 20 1

.....................AGAGCTTTCTTCAGTCCACTCA.................................................................................................................................................................... t2266440 22 1

..........................................ATGGGTGACAGTAAGATTCAAT............................................................................................................................................... t0112544 22 7

..........................................ATGGGTGACAGTAAGATTCAATT.............................................................................................................................................. t0332263 23 3

..........................................ATGGGTGACAGTAAGATTCAA................................................................................................................................................ t2388284 21 1

...........................................TGGGTGACAGTAAGATTCAATT.............................................................................................................................................. t0133703 22 6

...........................................TGGGTGACAGTAAGATTCAAT............................................................................................................................................... t0148717 21 6

................................................................TAGCTGCCGACTCATTCATCCA......................................................................................................................... t0004186 22 185

................................................................TAGCTGCCGACTCATTCATCC.......................................................................................................................... t0320646 21 3

................................................................TAGCTGCCGACTCATTCATC........................................................................................................................... t1328954 20 1

.................................................................AGCTGCCGACTCATTCATCCA......................................................................................................................... t0014414 21 58

.................................................................AGCTGCCGACTCATTCATCC.......................................................................................................................... t0173368 20 5

..................................................................................................................TCAGCAGATGAGTGAATGATG........................................................................ t1862972 21 1

...................................................................................................................CAGCAGATGAGTGAATGATGC....................................................................... t3250493 21 1

.......................................................................................................................AGATGAGTGAATGATGCGGGA................................................................... t3106298 21 1

............................................................................................................................AGTGAATGATGCGGGAGACAA.............................................................. t0002358 21 308

............................................................................................................................AGTGAATGATGCGGGAGACAAAT............................................................ t0002517 23 288

............................................................................................................................AGTGAATGATGCGGGAGACAAA............................................................. t0007943 22 104

............................................................................................................................AGTGAATGATGCGGGAGACA............................................................... t0010914 20 77

............................................................................................................................AGTGAATGATGCGGGAGACAAATT........................................................... t0053894 24 15

............................................................................................................................AGTGAATGATGCGGGAGAC................................................................ t0126727 19 7

............................................................................................................................AGTGAATGATGCGGGAGACAAATTG.......................................................... t2175155 25 1

.............................................................................................................................GTGAATGATGCGGGAGACAA.............................................................. t0410444 20 2

.............................................................................................................................GTGAATGATGCGGGAGACAAAT............................................................ t0893536 22 1

................................................................................................................................AATGATGCGGGAGACAAATTGA......................................................... t1955591 22 1

................................................................................................................................AATGATGCGGGAGACAAATTGAATC...................................................... t2180730 25 1

..................................................................................................................................TGATGCGGGAGACAAATTGAATC...................................................... t0315359 23 3

..................................................................................................................................TGATGCGGGAGACAAATTGAAT....................................................... t3318361 22 1

....................................................................................................................................ATGCGGGAGACAAATTGAATCT..................................................... t3181677 22 1

.......................................................................................................................................CGGGAGACAAATTGAATCTTA................................................... t1763817 21 1

...................................................................................................................................................TGAATCTTAAGTTTCCTGTAC....................................... t1935004 21 1

............................................................................................................................................................AGTTTCCTGTACTTGGACTGA.............................. t0973231 21 1

..............................................................................................................................................................TTTCCTGTACTTGGACTGAAG............................ t0611868 21 2

...................................................................................................................................................................TGTACTTGGACTGAAGGGAGC....................... t0262010 21 3

...................................................................................................................................................................TGTACTTGGACTGAAGGGAG........................ t0974908 20 1

....................................................................................................................................................................GTACTTGGACTGAAGGGAGC....................... t1278955 20 1

.......................................................................................................................................................................CTTGGACTGAAGGGAGCTCCCT.................. t0254594 22 4

.......................................................................................................................................................................CTTGGACTGAAGGGAGCTCCC................... t0490784 21 2

.......................................................................................................................................................................CTTGGACTGAAGGGAGCT...................... t3388238 18 1

........................................................................................................................................................................TTGGACTGAAGGGAGCTCCCTT................. t0021786 22 39

........................................................................................................................................................................TTGGACTGAAGGGAGCTCCCT.................. t0024064 21 35

........................................................................................................................................................................TTGGACTGAAGGGAGCTCCC................... t0106274 20 8

........................................................................................................................................................................TTGGACTGAAGGGAGCTCCCTTTT............... t2794714 24 1

**3. gma-MIR394a**

>gma-MIR394a(from 116SS)

ATCATGAGGGTTTAGCAAAGTGTTGCTAACAGAGTTTATTGGCATTCTGTCCACCTCCACTTCCTACTCTCTCTCTCTCTCTCTCTACCCATGAATATGGAGGTGGGCATACTGTCAACTGAGCTCTGTTGGCTACACTTTGTAAAGCCTTCATGAT gma-MIR394a 157

(((((((((((((.(((((((((.((((((((((((((((((((.(.((((((((((((.(((.............................)))..)))))))))))).).)))))).)))))))))))))).))))))))).))))))))))))) structure -78.95

*************************************************************************************************************************AGCTCTGTTGGCTACACTTT**************** gma-miR394a 20

.................AAGTGTTGCTAACAGAGTTTA....................................................................................................................... t0004859 21 162

.................AAGTGTTGCTAACAGAGTTT........................................................................................................................ t0680359 20 2

......................................TTGGCATTCTGTCCACCTCC................................................................................................... t0070165 20 12

................................................................................................ATGGAGGTGGGCATACTGTCAACT..................................... t0013159 24 64

....................................................................................................AGGTGGGCATACTGTCAACT..................................... t0005395 20 148

....................................................................................................AGGTGGGCATACTGTCAACTG.................................... t0006304 21 128

.........................................................................................................................AGCTCTGTTGGCTACACTTTG............... t0000238 21 2146

.........................................................................................................................AGCTCTGTTGGCTACACTTT................ t0002117 20 338

.........................................................................................................................AGCTCTGTTGGCTACACTT................. t0032664 19 25

.........................................................................................................................AGCTCTGTTGGCTACACT.................. t0060508 18 14

**4. gma-MIR2118a**

>gma-MIR2118a(from 116SS)

AAGGGAAAGGGAGAAGAGCTTGAGGAAGTGATGGGAGATGGGAGGGTCGGTAAAGAATATATCTGAGACTCGACTCAATCTCGATCTCTCTCAGTGTTGTGTTGTTTTGTTTATCCTTTTGCCGATTCCACCCATTCCTATGATTTCCTTCGGTTCCTCTCTTTCCACTC gma-MIR2118a 170

...((((((((((..(((((.((((((((.(((((((.((((.((((((((((((((((((.((((((.((((.......))))....))))))...)))))).............))))))))).))).))))))))))).)))))))).))))))))))))))).... structure -73.11

**********************************************************************************************************************TTGCCGATTCCACCCATTCCT******************************* gma-miR2118a 21

..........GAGAAGAGCTTGAGGAAGTGATG......................................................................................................................................... t0016787 23 50

..............................ATGGGAGATGGGAGGGTCGGTA...................................................................................................................... t0012538 22 67

...............................TGGGAGATGGGAGGGTCGGTA...................................................................................................................... t0046472 21 18

...............................TGGGAGATGGGAGGGTCGGTAA..................................................................................................................... t0058796 22 14

................................GGGAGATGGGAGGGTCGGTAAA.................................................................................................................... t0005645 22 142

................................GGGAGATGGGAGGGTCGGTAA..................................................................................................................... t0062103 21 13

.................................GGAGATGGGAGGGTCGGTAAAG................................................................................................................... t0000060 22 9703

.................................GGAGATGGGAGGGTCGGTAAA.................................................................................................................... t0000220 21 2295

.................................GGAGATGGGAGGGTCGGTAA..................................................................................................................... t0006204 20 130

.................................GGAGATGGGAGGGTCGGTA...................................................................................................................... t0008996 19 92

.................................GGAGATGGGAGGGTCGGTAAAGA.................................................................................................................. t0052819 23 16

.................................GGAGATGGGAGGGTCGGTAAAGAA................................................................................................................. t0063147 24 13

.....................................................................................................................TTTGCCGATTCCACCCATTCCTA.............................. t0154865 23 6

......................................................................................................................TTGCCGATTCCACCCATTCCTA.............................. t0000061 22 9480

......................................................................................................................TTGCCGATTCCACCCATTCCT............................... t0012977 21 65

......................................................................................................................TTGCCGATTCCACCCATTCC................................ t0021784 20 39

......................................................................................................................TTGCCGATTCCACCCATTCCTAT............................. t0034518 23 24

.......................................................................................................................TGCCGATTCCACCCATTCCTA.............................. t0044740 21 18

............................................................................................................................................TGATTTCCTTCGGTTCCTCT.......... t0186005 20 5

............................................................................................................................................TGATTTCCTTCGGTTCCTC........... t0349632 19 3

............................................................................................................................................TGATTTCCTTCGGTTCCTCTC......... t0663368 21 2

.............................................................................................................................................GATTTCCTTCGGTTCCTCTC......... t2503155 20 1

**E. Examples where the most abundant sequences were different from the annotated miRNA** (only sequences with more than 10 reads are shown except the most abundant variants and the annotated miRNA compared, the sequences that was the most abundant variants on the two arm of pre-miRNAs were shown in red,and the annotated miRNAs were shown in pink )

**1. gma-MIR160d**

>gma-MIR160d(from 116RL)

TGCCTGGCTCCCTGTATGCCATTTGTAGACCCCATTACAAAGGTGATGGCCTTAGCAAATGGCGTATGAGGAGTCATGCATGCTGTGTTTT gma-MIR160d 91

(((.(((((((..(((((((((((((.((..(((((((....)))))))..)).)))))))))))))..))))))).)))........... structure -44.60

TGCCTGGCTCCCTGTATGCC*********************************************************************** gma-miR160d 20

TGCCTGGCTCCCTGTATGCCA...................................................................... t0003305 21 606

TGCCTGGCTCCCTGTATGCC....................................................................... t0340193 20 3

.............................................................GCGTATGAGGAGTCATGCATG......... t0026379 21 52

**2. gma-MIR2109**

>gma-MIR2109(from 116RL)

GGTGCGAGTGTCTTCGCCTCTGAGAGAGATACTATGAGATCTCAAGCCTCGGAGGCGTAGATACTCACACC gma-MIR2109 71

((((.((((((((.((((((((((.(((((........)))))....)))))))))).)))))))).)))) structure -41.80

**TGCGAGTGTCTTCGCCTCTG************************************************* gma-miR2109 20

..TGCGAGTGTCTTCGCCTCTGA................................................ t0001052 21 1747

..TGCGAGTGTCTTCGCCTCT.................................................. t0020679 19 71

..TGCGAGTGTCTTCGCCTCTG................................................. t0135066 20 8

..................................................GGAGGCGTAGATACTCACACC t0003940 21 502

**3. gma-MIR482b**

>gma-MIR482b(from 116RL)

GGTATGGGGGGATTGGGAAGGAATATCCATAAGCAAAATATGCTATTTCTTCCCTACACCTCCCATACC gma-MIR482b 69

(((((((((((...(((((((((((..((((.......)))).)))))))))))....))))))))))) structure -35.80

**TATGGGGGGATTGGGAAGGAAT********************************************* gma-miR482b 22

..TATGGGGGGATTGGGAAGGAA.............................................. t0000324 21 4390

..TATGGGGGGATTGGGAAGGA............................................... t0000428 20 3556

..TATGGGGGGATTGGGAAGGAAT............................................. t0001585 22 1214

..TATGGGGGGATTGGGAAGG................................................ t0038683 19 33

...ATGGGGGGATTGGGAAGGA............................................... t0008719 19 206

...ATGGGGGGATTGGGAAGGAA.............................................. t0020766 20 70

...ATGGGGGGATTGGGAAGGAAT............................................. t0027086 21 51

...............................................TCTTCCCTACACCTCCCATACC t0000996 22 1838

...............................................TCTTCCCTACACCTCCCATAC. t0083785 21 14

**4. gma-MIR171e**

>gma-MIR171e(from 116RL)

ACATGGGGATGTTGGACGGTTCAATCAAATCAAATCTCCTAATGGCTGGGTCCTTTGGTATGATTGAGCCGTGCCAATATCAAATCCTGC gma-MIR171e 90

.((.(((((((((((((((((((((((.((((((...((((.....))))...)))))).)))))))))))).))))))))...))))). structure -39.60

************************************************************TGATTGAGCCGTGCCAATATC********* gma-miR171e 21

........ATGTTGGACGGTTCAATCAAA............................................................. t0016945 21 91

.........TGTTGGACGGTTCAATCAAA............................................................. t0001482 20 1291

.........TGTTGGACGGTTCAATCAAAT............................................................ t0039380 21 32

.........TGTTGGACGGTTCAATCAA.............................................................. t0049477 19 25

............................................................TGATTGAGCCGTGCCAATATC......... t0020326 21 72

**5. gma-MIR390c**

>gma-MIR390c(from 116RL)

TGTAAAGCTCAGGAGGGATAGCGCCATGGATGATCTCTTCTCCACTCTTGATCTTCTCTTGCGCTATCCATCCTGAGTTTCATGGCTTCT gma-MIR390c 90

.((.((((((((((.(((((((((...(((.((((..............)))).)))...))))))))).)))))))))).))....... structure -38.54

*************************************************************CGCTATCCATCCTGAGTTTC********* gma-miR390c 20

....AAGCTCAGGAGGGATAGCGCC................................................................. t0000832 21 2147

....AAGCTCAGGAGGGATAGCGC.................................................................. t0069038 20 17

....AAGCTCAGGAGGGATAGCGCCA................................................................ t0095357 22 12

.....AGCTCAGGAGGGATAGCGCC................................................................. t0019763 20 75

.............................................................CGCTATCCATCCTGAGTTTCA........ t0024541 21 57

.............................................................CGCTATCCATCCTGAGTTTC......... t0125778 20 9

**6. gma-MIR159d**

>gma-MIR159d (from 116RL)

GGGTGAATTGAGCTGCTTAGCTATGGATCCCACAGTTCTACCCATCAATAAGTGCTTTTGTGGTAGTCTTGTGGCTTCCATATCTGGGGAGCTTCATTTGCCT gma-MIR159d 103

(((..(((.(((((.(((((.((((((..((((((..(((((((..............)).)))))..))))))..)))))).))))).))))).)))..))) structure -47.24

**********AGCTGCTTAGCTATGGATCCC************************************************************************ gma-miR159d 21

..........AGCTGCTTAGCTATGGATCCC........................................................................ t0048379 21 25

..........................................................................CTTCCATATCTGGGGAGCTTC........ t0013720 21 118

...........................................................................TTCCATATCTGGGGAGCTTC........ t0069676 20 17
